# A versatile antibody capture system drives specific in vivo delivery of mRNA-loaded lipid nanoparticles

**DOI:** 10.1038/s41565-025-01954-9

**Published:** 2025-08-04

**Authors:** Moore Z. Chen, Daniel Yuen, Victoria M. McLeod, Ken W. Yong, Cameron H. Smyth, Bruna Rossi Herling, Thomas. J. Payne, Stewart A. Fabb, Matthew J. Belousoff, Azizah Algarni, Patrick M. Sexton, Christopher J. H. Porter, Colin W. Pouton, Angus P. R. Johnston

**Affiliations:** 1 Drug Delivery, Disposition and Dynamics, Monash Institute of Pharmaceutical Sciences, Monash University, Parkville, Victoria Australia; 2 Drug Discovery Biology, Monash Institute of Pharmaceutical Sciences, Monash University, Parkville, Victoria Australia; 3 ARC Centre for Cryo-electron Microscopy of Membrane Proteins, Monash Institute of Pharmaceutical Sciences, Monash University, Parkville, Victoria Australia

**Keywords:** Nanoparticles, Drug delivery

## Abstract

Efficient and precise delivery of mRNA is critical to advance mRNA therapies beyond their current use as vaccines. Lipid nanoparticles (LNPs) efficiently encapsulate and protect mRNA, but non-specific cellular uptake may lead to off-target delivery and minimal delivery to target cells. Functionalizing LNPs with antibodies enables targeted mRNA delivery, but traditional modification techniques require complex conjugation and purification, which often reduces antibody affinity. Here we present a simple method for capturing antibodies in their optimal orientation on LNPs, without antibody modification or complex purification. This strategy uses an optimally oriented anti-Fc nanobody on the LNP surface to capture antibodies, resulting in protein expression levels more than 1,000 times higher than non-targeted LNPs and more than 8 times higher than conventional antibody functionalization techniques. These precisely targeted LNPs showed highly efficient in vivo targeting to T cells, with minimal delivery to other immune cells. This approach enables the rapid development of targeted LNPs and has the potential to broaden the use of mRNA therapies.

## Abstract (web summary)

A mix-and-go system enables the rapid prototyping of antibody formulations, allowing control of their orientation on the surface of LNPs for specific cell targeting, significantly improving the ex vivo and in vivo deliveries of mRNA.

## Main

There is growing interest and an urgent need to develop precise, controlled and cost-effective systems to deliver therapeutic messenger RNA (mRNA)^1^. The successful deployment of two mRNA/lipid nanoparticle (LNP) vaccines to combat the COVID-19 pandemic has demonstrated the potential of LNPs to effectively deliver large synthetic mRNA payloads in vivo^2,3^. However, the low delivery efficiency and low specificity of current LNP formulations limits their use. Passive targeting of LNPs is generally achieved by manipulating lipid formulation (for example, different lipids or compositions) to achieve accumulation in the desired organs or cells^4^. Different charged lipids recruit different serum proteins to LNPs, resulting in altered in vivo biodistribution^5–7^. Library screens of hundreds of LNP formulations increased our ability to control the biodistribution of different LNPs in vivo^7^; however, this approach remains labour intensive and lacks a clear understanding of the specific cells targeted.

**Fig. 1:**
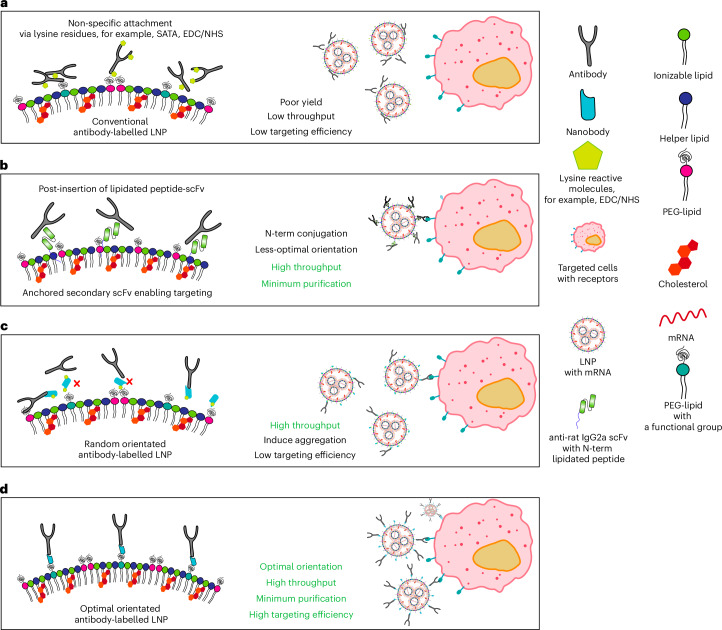
The TP1107 capture system attaches antibodies to LNPs in their optimal orientation. **a**, Schematics comparing conventional antibody conjugation. Typically, antibodies are conjugated to nanoparticles by randomly reacting succinimidyl esters with the naturally occurring lysine residues, which compromises the activity of the antibodies as they are randomly oriented on the surface of the nanoparticle. **b**, ASSET post-insertion of lipidated scFv. Antibodies can be captured on the surface of LNPs using an engineered anti-rat IgG2a scFv with an N-term modification with a lipidated peptide. **c**, Anti-mouse/anti-rat IgG1 nanobody TP1107 coupled via NHS-azide to lysine residues allows for high throughput evaluation of antibodies; however, the antibodies are still randomly oriented. **d**, Our optimally oriented TP1107 captures antibodies in their optimal orientation, maximizing the binding efficiency and enabling the simple and rapid screening of antibodies. These cartoons present an idealized representation of the antibody capture systems, and other factors such as flexibility of the PEG linker and topology of the nanoparticle surface influence the actual 3D structure on the surface of the particle.

Active targeting can be achieved by using targeting ligands such as antibodies or small-molecule ligands^8–11^. CD5-targeted LNPs that effectively produce anti-fibrotic chimeric antigen receptor T cells were recently reported^12^. The same approach was adapted for in vivo hematopoietic stem cell targeting with a CD117 (c-Kit)-targeted LNP, which showed effective in vivo stem cell editing and near-complete correction of hematopoietic sickle cells in vitro and the successful delivery of pro-apoptotic p53 upregulated modulator of apoptosis mRNA in vivo via targeted mRNA/LNP^13^. Although greatly improving the specificity of LNP delivery, conventional techniques for immobilizing antibodies onto particles affect the antibody binding affinity and often require complex and time-consuming purification protocols to isolate the targeted particles. The widely used succinimidyl ester or 1-ethyl-3-(3-dimethylaminopropyl)carbodiimide (EDC)/N-hydroxysuccinimide (NHS) conjugation chemistries rely on reactions with primary amines on lysine amino acids. Coupling via lysine residues results in antibodies that are randomly oriented on the nanoparticle surface (Fig. 1a) and can inactivate the antigen recognition domains. Previous work has shown that this attachment strategy produces notably poorer cell binding compared with antibodies that are conjugated via a specific site^14,15^. To enable greater control over the antibody orientation, disulfide bonds in the hinge region can be reduced to enable coupling of the reduced cysteines with maleimide groups. Although this can unify the orientation, the point of attachment is invariant and unlikely to orient the antibody in the optimal orientation. This approach also requires a precise control of the reducing conditions to ensure only disulfide bonds within the antibody’s hinge region are reduced. Failure to control these conditions can lead to the reduction of all disulfide bonds, resulting in antibody inactivation and aggregation^16,17^.

To overcome the limitations of chemical conjugation, non-covalent interactions can be used to attach antibodies onto LNPs. An innovative and flexible method for the antibody-targeted delivery of LNPs (ASSET) has been reported (Fig. 1b)^18^. Here an anti-rat IgG2a single-chain variable fragment (scFv) was immobilized onto the surface of the LNP via peptide lipidation, which served as a modular platform to capture rat IgG2a antibodies for active cell targeting^8,19^. Although this process eliminated the need to chemically modify antibodies and allowed for efficient and simple coupling to the LNP surface, the N-terminal lipidation of the anti-IgG2a scFv results in suboptimal antibody orientation on the LNP surface^20^. This leads to less-efficient cell targeting and restricts the ASSET system to capture a limited range of antibodies.

To overcome this problem, fine control over the protein conjugation site can be achieved through the incorporation of synthetic amino acids, as any residue in a recombinant protein can be substituted using stop-codon reassignment^21^. This allows site-specific conjugation for a controlled orientation of the protein on the surface of the particle^22^. One disadvantage of this approach is the need for a specially engineered bacterial strain and transfer RNA, which can lead to lower yields of protein.

Here we have developed a simple antibody capture system that requires no modification of the antibody, and ensures that the antibodies are attached onto the LNP in an orientation that increases binding to the target by eightfold compared with conventional antibody capture methods (for example, the widely used succinimidyl ester or EDC/NHS conjugation chemistries). We achieved this by modifying the LNPs with an antibody-capturing nanobody, TP1107 (ref. ^23^; Fig. 1d). To ensure the antibodies are captured on the surface of LNP with an orientation that maximizes binding to the target receptor, we used transmission electron microscopy (TEM) to determine the position and orientation of TP1107 binding to a mouse IgG1. Using this structure, we identified the optimal position in TP1107 for conjugation to the LNP surface, and used the site-specific incorporation of an azide-bearing synthetic amino acid (*p*-azido-phenylalanine (azPhe)) to allow insertion into the LNP. Due to the high affinity binding of TP1107 to the Fc domain of IgG1, antibodies can be simply added to the TP1107-functionalized LNPs with no requirement for further purification. We demonstrated that optimally oriented antibodies improved LNP binding and the delivery of mRNA more than 1,000 times compared with unmodified LNPs, as well as eight times more than conventional antibody modification. We also demonstrated that this technique can be used to rapidly screen a range of antibodies for the optimal specificity of the LNPs and improved expression of the mRNA. Using these actively targeted LNPs, we successfully transfected specific cell populations of primary human peripheral blood mononuclear cells (PBMCs) ex vivo, with minimal off-target expression. Finally, we demonstrated a highly efficient in vivo systemic targeting of T cells, with minimal binding to other immune cells. This approach allows for the rapid development of active targeted LNPs and holds the potential to expand the use of mRNA therapies.

## Rational design and expression of a nanobody to capture antibodies with optimal orientation

Critical to engineering a nanobody that captures antibodies in the optimal orientation is understanding the site at which the nanobody binds to the antibody. Previous work established that TP1107 nanobody binds the Fc portion of mouse IgG1 (ref. ^23^). To determine the site of nanobody binding, we used negative-stain TEM to image the protein complex. A 2:1 ratio of TP1107 and mouse anti-human transferrin receptor (clone OKT9) monoclonal antibody (mTfR) was negatively stained. Single particles were picked and extracted using RELION and two-dimensional classification was performed (Supplementary Fig. 1a)^24^. The two-dimensional class averages of the complex reveals a Y-shaped IgG structure along with two additional densities at the terminal ends of the Fc region, corresponding to two nanobodies bound to the antibody (Supplementary Fig. 1b).

The low-resolution electron microscopy image suggests the ‘side-on’ binding of TP1107 nanobody to the Fc domain of the IgG structure. On the basis of being (1) at a location distal from the predicted binding domain, (2) at a site outside the β-sheets or α-helices and (3) positioned on the protein’s outer surface that is probably to be sterically accessible, the Gln15 position is a promising site to conjugate TP1107 onto a nanoparticle surface for optimal antibody capture (Supplementary Fig. 1c, blue). Therefore, TP1107 with an azido-phenylalanine at Gln15 (TP1107_optimal_) was produced using a *Methnocaldococcus jannaschii*-derived orthogonal transfer RNA/synthetase pair^25^ and a genomically recoded *Escherichia coli* host^B-95.ΔA^ (ref. ^26^). We also expressed TP1107 without azido-phenylalanine and performed a random lysine modification with NHS-azide (TP1107_random_; Fig. 1c). A click-reactive fluorescent probe, DBCO-Cy5, was used to determine the efficiency of azide incorporation in these nanobodies. The degree of labelling for each batch of TP1107_optimal_ and TP1107_random_ was consistently ~0.5; however, due to the random nature of NHS modification, some of the randomly modified nanobodies were modified with multiple azide groups.

## LNP antibody capture system

**Fig. 2:**
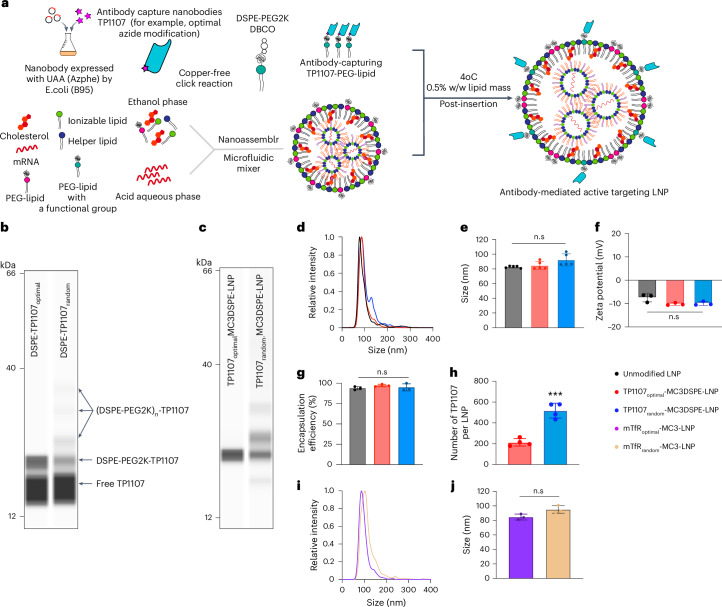
Formulation of LNPs that efficiently capture antibodies in their optimal orientation. Post-insertion of TP1107 into LNPs, stable particles are formed, which efficiently capture antibodies. **a**, Overview of the process to generate TP1107 LNP. **b**, ProteinSimple JESS Western assay showing lipid/nanobody conjugates as DSPE-TP1107_optimal_ and DSPE-TP1107_random_. **c**, ProteinSimple JESS Western assay showing lipid/nanobody conjugates inserted into MC3/DSPE LNP after purification with an ultrafiltration device. Experiments shown in **b** and **c** were repeated three times independently with similar results. **d**–**g**, Size distribution (**d**); mode size (*n* = 5 independent LNP preparation, *P* = 0.0514; **e**); zeta potential (*n* = 3 independent LNP preparation, *P* = 0.0524; **f**); and encapsulation efficiencies of unmodified LNP, TP1107_optimal_-MC3/DSPE LNP and TP1107_random_-MC3/DSPE LNP (*n* = 3 independent LNP preparations, *P* = 0.4096; **g**). **h**, Number of TP1107_optimal_ or TP1107_random_ per LNP from *n* = 4 different batches of LNPs, *P* = 0.0004. **i**,**j**, Size distribution (**i**) and mode size (**j**) of mTfR_optimal_-MC3 LNP and mTfR_random_-MC3 LNP (*n* = 3 independent LNP preparations, *P* = 0.0512). Data points represent each measurement and the error bars represent s.d. n.s represents no significant differences, ***P < 0.001, ****P < 0.0001. We used an unpaired *t*-test: two-tailed for **h** and **j**, Dunnett’s multiple comparisons test for **g** and one-way analysis of variance with post hoc Tukey’s test for **e** and **f** (we compared means of columns with the means of every other column). Source data

Next, TP1107_optimal_ and TP1107_random_ were modified with a DSPE-PEG_2000_-DBCO lipid (Fig. 2a). The lipid and TP1107 were conjugated at a 2:1 molar ratio (DBCO:azide). 12.2% of TP1107_optimal_ was coupled to a single DSPE-PEG_2000_-DBCO, as evidenced by the single band at 22 kDa using a capillary western assay (Fig. 2b). In comparison, 6.6% of the TP1107_random_ was conjugated to a single DSPE-PEG_2000_, with three additional bands, at 26 kDa (4.2%), 32 kDa (2.3%) and 36 kDa (2.1%), indicating conjugation to two, three and four lipids, respectively (Fig. 2b).

Typically, LNP formulations comprise four primary components: (1) ionizable lipids—such as the clinically approved DLin-MC3-DMA (MC3) or SM102; (2) helper lipids, including DSPC, DOPE and cholesterol, which provide structural support; (3) PEG-lipids that enhance particle stability; and (4) mRNA. In this study, we examine two structurally distinct ionizable lipids (MC3 and SM102) and two PEG-lipids, with differing carbon chain lengths of their lipophilic anchors (Supplementary Fig. 3). Specifically, DMG-PEG_2000_ has a shorter C14 chain, making it more prone to dissociation in vivo, whereas DSPE-PEG_2000_, with its longer C18 chain, remains more stably associated.

The hydrodynamic diameter of MC3/DSPE LNPs measured by NanoSight (NTA) was 83 nm (Fig. 2d,e and Supplementary Table 1). To allow nanobody insertion, the DBCO-PEG_2000_-DSPE-TP1107 mixture was incubated with the LNPs at 0.5% w/w (DBCO-PEG_2000_-DSPE versus total lipid) for 48 h at 4 °C. Unincorporated DSPE-PEG_2000_-TP1107 was removed by 100 kDa molecular weight cut-off (MWCO) ultrafiltration. Western blotting showed that after purification, no unmodified nanobodies were detected (Fig. 2c). TP1107_optimal_ exhibited a single band, corresponding to nanobody modified with a single DBCO-PEG_2000_-DSPE, whereas TP1107_random_ exhibited four bands corresponding to 1 (52.5%), 2 (25.9%), 3 (14.3%) and >4 (7.3%) PEG_2000_-DSPE modifications. These multiple bands are consistent with the pattern observed for DSPE-PEG_2000_-TP1107_random_ (Fig. 2b).

Next, we assessed the basic physical properties of the TP1107-modified LNPs. LNP size was measured using NanoSight, with the diameter of the TP1107_optimal_-MC3/DSPE LNP at 85 nm and that of TP1107_random_-MC3/DSPE LNP at 92 nm, which was similar to the size of unmodified LNPs (Fig. 2d,e and Supplementary Table 1). We did not expect to see a large increase in the diameter of LNPs on functionalization, as the small dimensions of the nanobody (2 × 2 × 4 nm^3^) are within the uncertainty of the NanoSight measurement. TP1107_random_-MC3/DSPE LNP also showed a second population sized at 132 nm. The NanoSight measurements were made on the same batch of LNPs before and after the insertion of TP1107_optimal_ and TP1107_random_. Therefore, the appearance of the second population in the TP1107_random_ LNPs can be attributed to LNP aggregation. TP1107_optimal_-MC3/DSPE LNP showed the same size distribution to the unmodified LNP, with no particle aggregation detected. Attaching TP1107 did not alter the surface charge or the encapsulation efficiency of the LNPs (Fig. 2e,f and Supplementary Tables 1 and 2). To determine the number of nanobodies attached per particle, the particle concentration was determined using NanoSight (Supplementary Table 3) and the concentration of nanobody interpolated from the calibrated western analysis (Supplementary Table 3). From the ratio of these numbers, the average number of nanobodies per LNP was estimated to be ~200 for TP1107_optimal_-MC3/DSPE LNP and 400-600 for TP1107_random_-MC3/DSPE LNP (Fig. 2h). Although the number of nanobodies per LNP differs when the same amount of TP1107_optimal_ and TP1107_random_ is added, the number of antibodies per LNP can be controlled by varying the amount of antibody added (Fig. 4). Collectively, these results indicate that the optimally oriented TP1107-PEG_2000_-DSPE is efficiently incorporated into the LNPs without impacting the size of the particles, whereas the random conjugation method resulted in TP1107 being labelled with more than one DSPE-PEG_2000_-DBCO and caused a small amount of particle aggregation.

Functionalization of LNPs with a targeting antibody was achieved by simply adding the antibody to the TP1107-functionalized particles at a twofold excess of TP1107 to the antibody. The high affinity of TP1107 to the Fc domain of mouse IgG1 implies that under these conditions, all antibodies were captured (Supplementary Fig. 4). TP1107_optimal_-MC3/DSPE LNP modified with anti-transferrin receptor mouse IgG1 (mTfR) formed LNPs with a single population with a diameter of 85 ± 4 nm for mTfR_optimal_-MC3 LNP. The size of the LNPs with randomly captured mTfR was similar (95 ± 8 nm for mTfR_random_-MC3 LNP, *n* = 5). These results confirmed the ability to attach the antibody-capturing TP1107 nanobody onto the surface of LNPs, and that this modification did not significantly change the size, surface charge or the ability to efficiently encapsulate mRNA (Supplementary Tables 1 and 2). We additionally assessed an SM102 LNP formulation and observed similar results (Supplementary Table 1).

To test whether the nanobody:antibody complex on LNPs is stable in plasma, we incubated the antibody-modified LNPs in human plasma at 37 °C overnight. We observed no antibody detachment (Supplementary Fig. 4). This confirmed robust antibody capture on the surface of the LNPs and that the capture system is suitable as an active targeting strategy for mRNA delivery.

## Optimizing delivery to specific cells

**Fig. 3:**
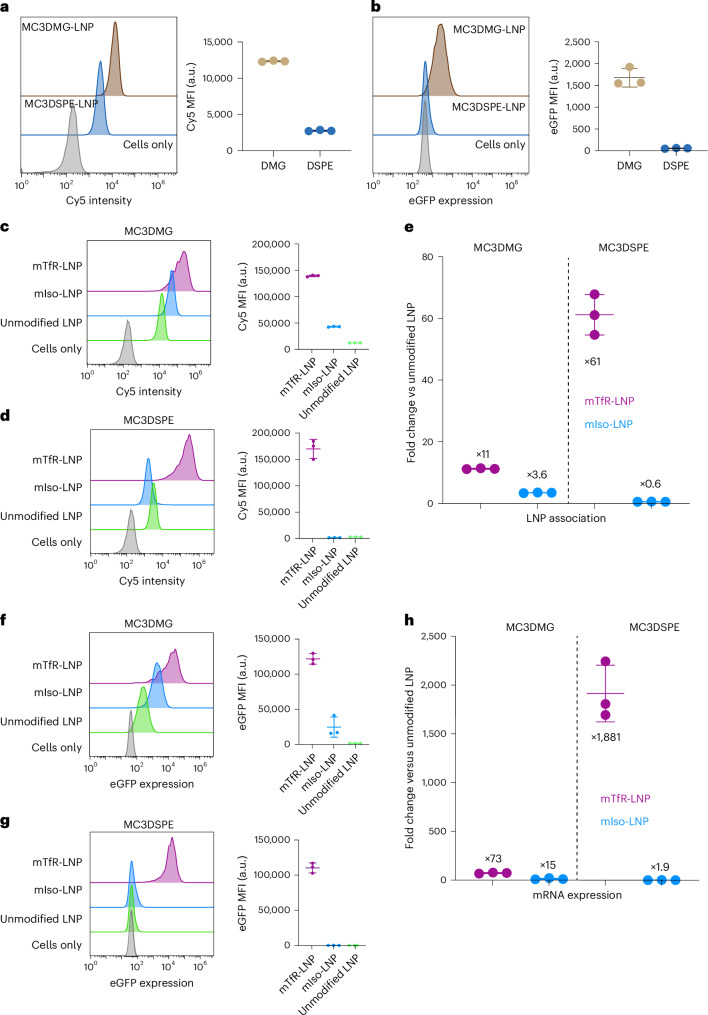
DSPE-PEG_2000_ LNPs limit non-specific cell binding and maintain high antibody-mediated binding. **a**, Cy5 MFI of Jurkat cells incubated with MC3/DMG LNP or MC3/DSPE LNP. **b**, eGFP expression level of Jurkat cells incubated with MC3/DMG LNP or MC3/DSPE LNP. **c**,**d**, Cy5 MFI of MC3/DMG LNP (**c**) and of MC3/DSPE LNP (**d**) incubated with human TfR-targeted LNP, isotype control and unmodified LNP with Jurkat cells. **e**,**f**, eGFP expression MFI of MC3/DMG LNP (**e**) and of MC3/DSPE LNP (**f**) incubated with human TfR-targeted LNP, isotype control and unmodified LNP with Jurkat cells. **g**,**h**, Calculated fold-change difference between targeted LNP versus unmodified LNP for LNP association (**g**) and mRNA expression (**h**) in treated Jurkat cells. Data are represented as mean ± s.d. (*n* = 3 replicate wells). Source data

To engineer an efficient actively targeted LNP system, the base LNP formulation should have minimal non-specific association with cells. For this purpose, we investigated two PEG-lipids: DMG-PEG_2000_ (used in the Moderna vaccine formulation) and DSPE-PEG_2000_ (with longer alkyl chains that may better anchor the PEG to the surface of the LNP). Both these PEG-lipids were formulated using two of the current clinically approved ionizable lipids (SM102 (used in the Moderna vaccine) and DLin-MC3-DMA (used in Onpattro)). LNPs were formulated with mRNA-encoding eGFP spiked with a Cy5-labelled oligo to assess the induced protein expression and association of LNPs to cells, respectively. LNP formulations were incubated with Jurkat cells for 24 h at an mRNA concentration of 0.5–1 ng µl^−1^. Untargeted MC3/DMG LNP showed more than fivefold non-specific association compared with untargeted MC3/DSPE LNP after 24 h (Fig. 3a). Untargeted MC3/DSPE LNP exhibited limited eGFP expression, whereas untargeted MC3/DMG LNP induced strong eGFP expression (Fig. 3b). Next, we used our TP1107 capture system to functionalize these LNPs with mTfR, which binds to human TfR that is expressed on the surface of Jurkat cells. A mouse IgG1 isotype control antibody (mIso) was used to assess the potential non-specific association induced by modifying the PEGylated LNP surface with proteins. Both mTfR-MC3/DMG LNPs and mTfR-MC3/DSPE LNPs showed significantly increased association with cells compared with unmodified LNPs (Fig. 3c,d). The association of both targeted formulations to cells was similar; however, the lower non-specific association of the mTfR-MC3/DSPE LNP resulted in a 61-fold increase in specific binding, compared with an 11-fold increase with mTfR-MC3/DMG LNP (Fig. 3e). eGFP expression from mRNA delivered by the LNPs followed a similar trend. mTfR-MC3/DMG LNPs and mTfR-MC3/DSPE LNPs showed high levels of eGFP expression (Fig. 3f,g). Again, the unmodified and mIso-MC3/DSPE LNPs maintained their low non-specific binding, with minimal eGFP expression. A more than 1,880-fold increase in eGFP expression was observed with mTfR-MC3/DSPE LNPs compared with unmodified MC3/DSPE LNPs. Although mTfR-MC3/DMG LNPs showed similar eGFP expression to mTfR-MC3/DSPE LNPs, DMG LNPs showed a lower 73-fold improvement compared with unmodified LNPs (Fig. 3h) due to the higher non-specific expression of eGFP.

To demonstrate that the antibody capture system is compatible with different LNP formulations, we also formulated DMG LNP and DSPE LNP with SM102 as the ionizable lipid rather than MC3. SM102 LNP formulations were incubated with Jurkat cells for 24 h at an mRNA concentration of 0.5 ng µl^−1^ (Supplementary Fig. 5). We observed similar trends with SM102 and MC3 LNPs, with both mTfR-SM102/DMG LNPs and mTfR-SM012/DSPE LNPs having significantly higher cell association (Cy5 mean fluorescence intensity (MFI); Supplementary Fig. 5a,b) and higher mRNA delivery (eGFP expression MFI; Supplementary Fig. 5c,d) compared with the isotype control and unmodified counterpart. Again, SM102/DMG LNP showed a higher non-specific association and transfection compared with SM102/DSPE LNP, which resulted in a lower fold change (Supplementary Fig. 5e,f). As observed in other studies comparing the effect of the ionizable lipid, all the SM102 formulations induced significantly higher protein expression than MC3 (ref. ^27^). Therefore, hereafter, we formulated all the LNPs with DSPE-PEG_2000_ as the PEG component.

**Fig. 4:**
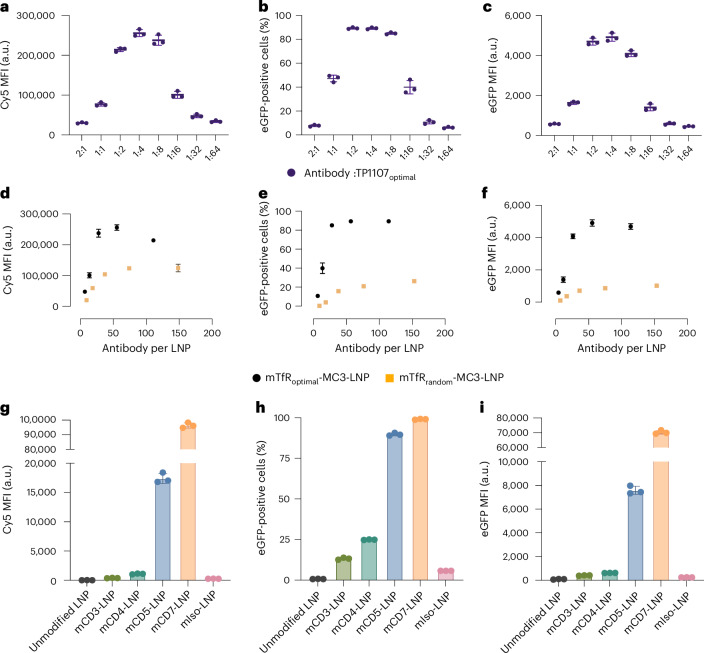
Optimally oriented antibodies show significantly higher cell binding and protein expression than randomly oriented antibodies. **a**–**c**, Cy5 MFI (**a**), percentage of eGFP-positive cells (**b**) and eGFP MFI (**c**) of Jurkat cells incubated with TP1107 _optimal_-MC3 LNP incubated with different ratios (64:1 to 1:2) of TP1107 versus mTfR over 4 h with 0.5 ng µl^−1^ of mRNA. **d**–**f**, Comparison of Cy5 MFI (**d**), percentage of eGFP-positive cells (**e**) and eGFP MFI (**f**) of Jurkat cells incubated with TP1107_optimal_-MC3 LNP or TP1107_random_-MC3 LNP functionalized with different numbers of mTfR (ranging from 7 to 148 antibodies per LNP) over 4 h at 0.5 ng µl^−1^. **g**–**i**, TP1107_optimal_-MC3 LNP functionalized with a panel of mAb (anti-human CD3, CD4, CD5, CD7 and isotype control) were incubated with Jurkat cells for 24 h: Cy5 MFI (**g**), percentage of eGFP-positive cells (**h**) and eGFP MFI (**i**). All data are represented as means and the error bars represent mean ± s.d. (*n* = 3). Data points represent replicate wells. Experiments have been repeated three times. Source data

To further tune the antibody capture on LNPs, we investigated how antibody density on the LNP affects cell binding and the efficiency of protein expression from the delivered mRNA. To determine the efficiency of antibody capture onto the LNPs, we used native polyacrylamide gel electrophoresis followed by western blot against the antibody to measure the amount of free antibody in solution after functionalization. Here 100 ng of mTfR_optimal_ or mTfR_random_ MC3/DSPE LNP with different ratios of antibody:TP1107 (1:64, 1:32, 1:16, 1:8, 1:4, 1:2, 1:1 and 2:1) were loaded into the gel. Free antibody can enter the gel, whereas LNPs and the antibodies captured on the surface of the LNPs remain trapped in the well. There was minimal detectable free antibody for antibody:TP1107 ratios of 1:64, 1:32, 1:16, 1:8, 1:4 or 1:2 (Supplementary Fig. 6), indicating that the antibody capture was quantitative, and that these LNPs could be used without any further purification. The 1:1 and 2:1 ratios showed increasing levels of free antibody. To test the cell binding and protein expression induced by the LNPs with different levels of antibody functionalization, we incubated mTfR_random_ LNPs or mTfR_optimal_ LNPs with Jurkat cells for 4 h without removing the unbound antibody. As expected, cell binding (Cy5 fluorescence) and eGFP expression increased as the amount of targeting antibody on the surface of the LNPs increased, with the maximum cell association and eGFP expression observed with ratios of 1:8, 1:4 and 1:2 (mTfR:TP1107 ratio). At the highest antibody:TP1107 ratios (1:1 and 2:1), the cell binding and eGFP expression significantly decreased (Fig. 4a–c and Supplementary Fig. 7a,b). This is probably due to the free antibody binding to Tf receptors on the cell surface and blocking binding of the targeted LNPs. Although it is possible to purify the targeted LNPs from the unbound antibodies, this increases the complexity of the functionalization process and probably results in lower yields of LNPs. From these data, we determined the 2:1 TP1107:antibody ratio as the optimal functionalization ratio, as it requires no further purification of the LNPs and exhibits significantly higher cell binding and higher protein expression than the unmodified control.

We then investigated whether the optimal orientation of TP1107 (TP1107_optimal_) showed superior cell binding and eGFP expression compared with TP1107_random_ with matching antibody number per LNP. mTfR_optimal_-MC3 LNP exhibited almost two times greater cell binding and more than five times higher eGFP expression compared with mTfR_random_-MC3 LNP (Fig. 4d–f).

We were interested to compare our antibody capture system to the strategy that is currently used widely to directly couple antibodies to the surface of LNPs^12^. One widely used linker is N-succinimidyl S-acetylthioacetate, which introduces protected sulfhydryl groups by reacting randomly with lysine residues on the antibody and subsequently reacted with maleimide-modified lipids^28^. In this work, we chose to use NHS-PEG_6_-azide, to ensure that the coupling chemistry was consistent with our nanobody capture system. Both linkers react randomly with lysine residues, and enable efficient coupling to the lipid. mTfR was reacted with NHS-PEG_6_-azide, which gave a degree of labelling of 0.5. Subsequently, DBCO-PEG_2000_-DSPE lipid was conjugated to the azide-modified antibody using the same protocol as the TP1107 lipid conjugation. The LNP and mTfR_Lysine_ mixture was then purified via size exclusion gel chromatography. Then, cell binding and mRNA delivery of the mTfR_Lysine_-SM102 LNP was compared with mTfR_optimal_-SM102 LNP. Although both targeting methods induced 100% eGFP expression in Jurkat cells (Supplementary Fig. 8a), the mTfR-TP1107_optimal_ LNP showed a higher cell association (Supplementary Fig. 8b). More importantly, eGFP expression levels were more than eight times higher for our optimally oriented targeted LNP (Supplementary Fig. 8c). These results confirmed that mTfR_optimal_-SM102 LNP outperformed mTfR_Lysine_-SM102 LNP.

## TP1107 capture system allows rapid screening of a panel of targeting antibodies

To demonstrate the feasibility of our antibody capture system to rapidly screen a panel of different antibodies, we prepared TP1107_optimal_-MC3/DSPE LNP with antibodies against human CD3, CD4, CD5 and CD7, which are targets for drug delivery or chimeric antigen receptor T cell therapy^28–30^ due to their abundancy on T cells. First, we measured the surface expression level of these markers on Jurkat cells. There was limited CD3 expression; however, there was robust surface expression of CD4, CD5 and CD7 (Supplementary Fig. 9). We next investigated LNP association to the cells. mCD5 and mCD7 LNP showed higher levels of binding to Jurkat cells than mCD3 and mCD4 (Fig. 4g). Both mIso and unmodified LNPs showed similar levels of non-specific association. The association of mAb LNP to Jurkat cells followed a similar trend to that observed for free mAbs, indicating that our antibody capture method does not interfere with antibody–receptor binding. Interestingly, as observed for the mTfR LNPs, the increase in eGFP expression was even greater than the increase in cellular association for the targeted LNPs. Both mCD5 and mCD7 LNPs showed high expression levels with ~90–100% of the cells expressing eGFP after 24 h (Fig. 4h,i). Although the unmodified and isotype LNPs showed low levels of non-specific association to Jurkat cells, there was minimal eGFP expression.

## Targeting specific T cell receptors maximizes protein expression in human PBMCs

**Fig. 5:**
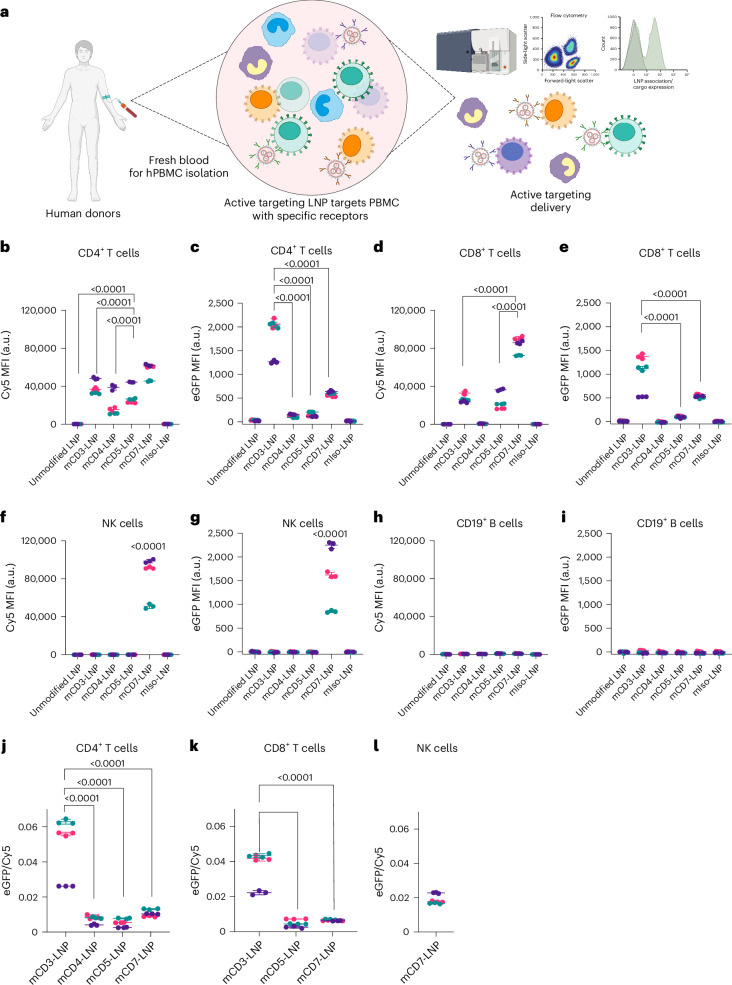
A screen of T cell-targeting antibodies in human PBMCs reveals that LNPs targeted to CD3 induces efficient protein expression in T cells. **a**, Schematic illustrating the process of collecting human PBMC and accessing the specific targeting ability of active targeting LNP system by flow cytometry. **b**–**i**, Human PBMC was incubated with a panel of mAb LNP (CD3, CD4, CD5, CD7, isotype control and unmodified LNP) at an mRNA concentration of 2 ng µl^−1^ for 24 h. CD4^+^ T cell (**b** and **c**), CD8^+^ T cells population (**d** and **e**), CD56^+^ NK cell population (**f** and **g**) and CD19^+^ B cell population (**h** and **i**). Panels **b**, **d**, **f** and **h** show LNP association (Cy5 MFI), panels **c**, **e**, **g** and **i** show the mRNA delivery (eGFP MFI) and panels **j, k** and **l** show the ratio between eGFP MFI/Cy5 of groups that have meaningful LNP association (Cy5 MFI > 3,000 and ratio > 0) of CD4^+^ T cells (**j**), CD8^+^ T cells (**k**) and NK cells (**l**). All data were collected from three donors (*n* = 3; depicted by green, pink and purple) and conducted in a triplicate well and shown as an independent dot. Data points represent means of each donor with three technical replicates and the error bars represent s.d. *P value is labelled in the figure and calculated using a two-way analysis of variance with post hoc Tukey’s test (compare row means with the main row effects). Panel **a** created with BioRender.com. Source data

To further demonstrate the capability of this targeted LNP system, we investigated the ability to target specific subsets of human PBMCs. PBMCs were isolated from the blood of healthy donors using Ficoll gradient purification (Fig. 5a). Human CD3-, CD4-, CD5- and CD7-targeted MC3 LNP with eGFP mRNA and Cy5-labelled oligos were incubated with PBMC for 24 h. Cells were then analysed by flow cytometry for LNP binding (Cy5 signal) and eGFP expression, using an antibody-phenotyping panel for identifying the subpopulation (Supplementary Fig. 10a). As expected, CD3-, CD4-, CD5- and CD7-targeted LNP showed strong binding towards CD4^+^ T cells, as these receptors are present on the cell surface (Fig. 5b and Supplementary Fig. 11a). Interestingly, although all CD4^+^ T cells had targeted LNPs bound, mCD3 LNPs showed lower CD4^+^ T cell association than mCD7 LNPs; however, the eGFP expression was significantly higher (Fig. 5c and Supplementary Fig. 11b). Furthermore, although mCD4 and mCD5 LNPs showed only slightly lower CD4^+^ T cell association than mCD3 and mCD7 (10–50% reduction in association), eGFP was significantly lower (90% reduction). A similar phenomenon was observed for CD8^+^ T cells (Fig. 5d,e and Supplementary Fig. 11c,d). mCD7 LNPs showed the highest association with CD8^+^ T cells; however, mCD3 LNPs showed significantly higher eGFP expression (Fig. 5e). This illustrates the role of the receptor in mediating the uptake and delivery of mRNA and highlights the importance of screening for the optimal receptor. Only mCD7 LNPs showed significant binding to NK cells (Fig. 5f and Supplementary Fig. 11e), which resulted in a correspondingly high eGFP signal (Fig. 5g and Supplementary Fig. 11f). Both unmodified and mIso LNPs showed no detectable binding to T cells and NK cells. As expected, CD19^+^ B cells showed neither binding nor expression with either unmodified or targeted LNPs because of the lack of receptors on the surface (Fig. 5h,i and Supplementary Fig. 11g,h). To understand the relationship between LNP binding and eGFP expression, we ratioed the eGFP expression to the Cy5 binding signal for all the samples in which the Cy5 signal was significantly higher than the background signal (Fig. 5j,k,l). These results confirmed that LNPs targeted to CD3 are significantly more efficient at inducing protein expression^31–33^.

We were also interested to test if the superior targeting ability of our system was sustained when using a more potent ionizable lipid. CD3-, CD4-, CD5- and CD7-targeted LNPs as well as IgG1 control and unmodified LNPs were prepared with eGFP mRNA and SM102 as the ionizable lipid. Mirroring what we observed with the MC3 LNPs, CD3-, CD4-, CD5- and CD7-targeted LNPs induced a significant increase in eGFP expression in CD4^+^ T cells compared with the IgG1 isotype control (Supplementary Fig. 12a). In CD8^+^ T cells, the CD3-, CD5- and CD7-targeted LNPs but not CD4-targeted LNPs demonstrated increased eGFP expression (Supplementary Fig. 12b). mCD7 LNPs targeted both T cells and NK cells, which was consistent with mCD7-MC3 LNPs (Supplementary Fig. 12c). No off-targeted delivery to CD19^+^ B cells was observed with any of the antibody-targeted LNPs (Supplementary Fig. 12d). This indicates that the enhanced expression induced by our targeting system is probably applicable to a range of ionizable lipids.

To further demonstrate that our targeted LNP system can target different cell types, we investigated whether we could specifically induce expression in B cells by targeting the LNPs via the CD22 receptor. CD22, a surface antigen expressed on B lymphocytes, is a member of the sialic-acid-binding immunoglobulin-like lectins (Siglec) family. This receptor is known to undergo recycling from the cell surface to the endosomal compartment, making it a promising target for B cell-specific delivery systems^34,35^. The CD19^+^ B cell population showed a significant increase in eGFP MFI on treatment with mCD22 LNPs, with no expression detected in B cells when PBMCs were treated with mIso LNPs (Supplementary Fig. 13). Importantly, mCD22 LNPs did not induce eGFP expression in CD22^−^ cell populations (CD4^+^ T cells, CD8^+^ T cells and NK cells; Supplementary Fig. 13). This demonstrates that our targeted LNP system can be engineered to target specific cell types depending on the antibody captured on the LNP.

## Targeting circulating T cells in vivo

**Fig. 6:**
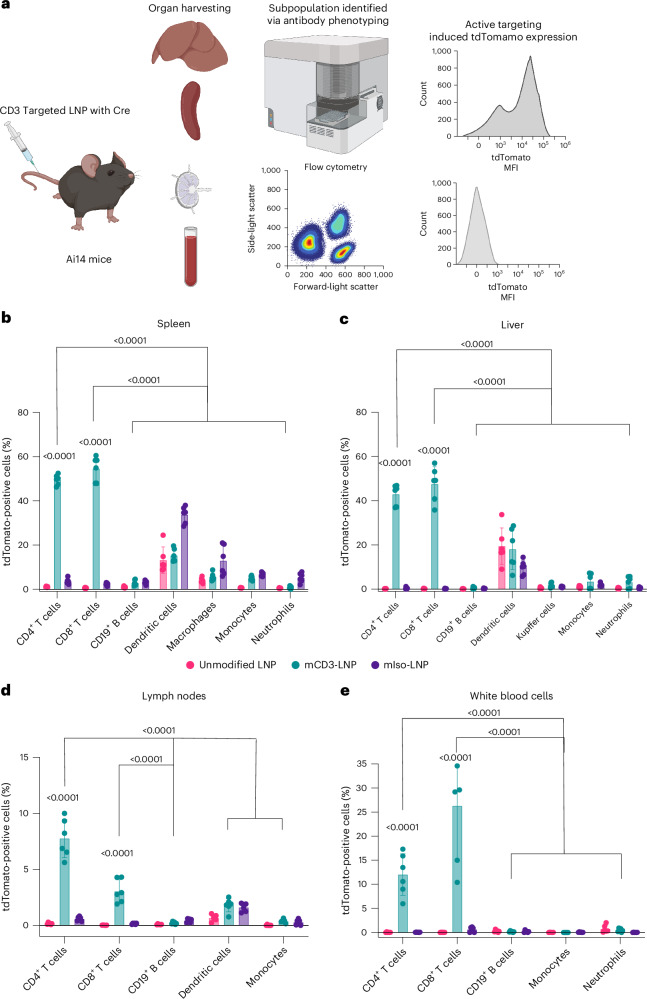
Anti-CD3 LNP-targeted T cells resident in spleen, liver, lymph nodes and white blood cells in vivo with minimal off-targeting effect. **a**, Schematic of Ai14 mice given 0.1 mg kg^−1^ of either CD3-targeted LNP, isotype control LNP and unmodified LNP with Cre mRNA intravenously. Immune cells were harvested from the spleen, liver, lymph nodes and circulating blood after 24 h. **b**–**e**, Murine splenocytes (**b**), murine liver immune cell population (**c**), murine lymph node immune cell population (**d**) and murine white blood cells (**e**) were isolated and enriched. The stained cells were analysed by flow cytometry to identify the percentage of tdTomato-positive cells in each subpopulation (CD4^+^ T cell, CD8^+^ T cell, dendritic cells, CD19^+^ B cells, monocytes, macrophage/Kupffer cells and neutrophils). *n* = 6 biologically independent mice in two separate cohorts. All data are means and the error bars represent s.d. *P* value is labelled in the figure and calculated by two-way analysis of variance with post hoc Tukey’s test (compare row means (compare cell means with others in its row and its column)). Panel **a** created with BioRender.com. Source data

Finally, to demonstrate that the highly specific T cell targeting we observed ex vivo translates in vivo, we administered CD3-targeted LNPs loaded with Cre mRNA into Ai14 mice. These mice have a loxP-flanked STOP cassette preventing the transcription of CAG promoter-driven tdTomato. When Cre recombinase is delivered to any cell in these mice, the STOP cassette is excised and a strong tdTomato expression is observed. This allows us to sensitively determine which immune cell population the Cre mRNA is delivered to in vivo. Twenty-four hours after the intravenous injection of 0.1 mg kg^−1^ of mRNA (or ~2 µg of mRNA per 20 g mouse), blood was collected via cardiac puncture, and the liver, spleen and lymph nodes (inguinal, iliac and cervical) were collected and purified to enrich the immune cells. The major immune cell populations were phenotyped (Supplementary Fig. 10b–e) and the percentage of tdTomato cells (Supplementary Fig. 10f) in each subpopulation was used to quantify the successful delivery of Cre mRNA (Fig. 6a).

We observed that mCD3 LNP induced a significant increase in tdTomato-positive T cells in the spleen (50% of CD4^+^ T cells and 54% CD8^+^ T cells), liver (43% of CD4^+^ T cells and 47.5% CD8^+^ T cells), lymph nodes (8% of CD4^+^ T cells and 3–4% of CD8^+^ T cells) and circulating blood (12% of CD4^+^ T cells and 26% of CD8^+^ T cells) compared with its mIso and unmodified LNP (below 4% across all organs; Fig. 6b–d). This result indicates that our targeted system can selectively target the cell populations expressing specific receptors in vivo. This highly specific targeting of our LNP system demonstrates its potential to expand mRNA delivery to therapeutic targets that are not accessible with passive LNP uptake. More importantly, our targeted LNPs did not increase non-specific delivery in bystander cells compared with its isotype control. To compare the performance of mCD3_optimal_ LNP and mCD3_lysine_ LNP, we also prepared and administered mCD3_lysine_ LNP to mice (Supplementary Fig. 14). Approximately 5% of T cells in the spleen, 10% in the liver, and less than 5% in both lymph nodes and white blood cells were tdTomato positive. Bystander cells, such as dendritic cells and macrophages, exhibited a similar proportion of tdTomato-positive cells (Supplementary Fig. 14). These results were probably due to the lower dosage used in this study (2 µg per mouse) compared with previous experiments that used 10 µg per mouse. This result indicated that our antibody-capturing targeted LNPs demonstrated notable advantages for in vivo targeted delivery compared with conventional antibody-targeted LNPs.

We also assessed the potential toxicity of this targeting system ex vivo. LNPs were incubated with 200 µl of whole blood for 24 h at a concentration of 2 ng µl^−1^. Plasma was then extracted and cytokine concentration was measured (Supplementary Fig. 15). All mAb-modified LNPs exhibited elevated levels of IL-1b, TNF and IL-1a compared with unmodified LNPs (Supplementary Fig. 15a–c). This stimulation is probably due to using mouse-derived antibody with human cells. mCD3 LNPs also exhibited increased IFNg (Supplementary Fig. 15d), although the other mAb-modified LNPs exhibited similar levels as unmodified LNPs, suggesting that IFNg is linked to the specific antibody rather than the LNP formulation. This anti-CD3-mediated activation was anticipated as others have shown that anti-CD3 antibodies can activate T cells by binding to the CD3e:TCR complex^36^.

For in vivo delivery, we found no significant weight loss on the delivery of mCD3 LNP compared with the current standard delivery formulation or phosphate-buffered saline (PBS) vehicle control (Supplementary Fig. 16a), which indicates that the addition of nanobody to the LNP is well tolerated. Although there was a slight increase in spleen weight in mice treated with mCD3 LNP (Supplementary Fig. 16b), histopathological evaluation of the spleen and liver 24 h post-treatment revealed no remarkable differences (Supplementary Fig. 16c). No liver toxicity was observed between the vehicle-treated groups by evaluating the alanine transaminase and aspartate transaminase concentrations in plasma at 6 h post-dose (Supplementary Figs. 16d and 1e).

A cytokine panel was measured in the plasma at 24 h post-dosing. Of the cytokines evaluated, only IL-4 and MCP-1 (CCL-2) were above the limit of detection (10 pg ml^−1^). All the four groups (vehicle (PBS), unmodified SM102-DSPE, unmodified SM102-DMG as the current common formulation and mCD3 LNP) showed no significant difference in IL-4 levels. However, the mCD3 LNP group exhibited a small increase in MCP-1 levels 24 h post-dosing (Supplementary Fig. 16f,g). This is consistent with other reports that show elevated levels of MCP-1 (CCL-2) after treatment with anti-CD3 in vivo^37^.

## Conclusions

We have developed a highly versatile antibody capture process that attaches antibodies to the surface of LNPs in their optimal orientation. Site-specific lipid conjugation to nanobodies facilitated self-insertion onto LNPs, streamlining assembly and permitting the rapid generation of receptor-targeted LNPs. This approach surpasses conventional antibody/LNP systems in orientation, efficiency and manufacturability. In vivo administration of CD3-targeted LNPs in Ai14 reporter mice confirmed efficient mRNA delivery to T cells, with minimal off-target delivery to other immune cells, consistent with in vitro and ex vivo results. We have shown that the selection of the surface receptor plays a significant role in the efficiency of protein expression. High receptor expression levels and high LNP association does not necessarily correlate to high levels of protein expression, and the ability of the targeted receptor to internalize is probably key to utility. These results are encouraging for the future development of non-vaccine mRNA therapeutics. As mRNA therapeutics evolve, precision delivery will be vital for maximizing efficacy and minimizing off-target effects. Our modular platform facilitates the rapid identification of optimal targets, accelerating therapeutic development. The methodology holds broad applicability for diverse nucleic acid delivery, supporting future non-vaccine mRNA applications.

## Methods

### Materials

#### mRNA synthesis

All in vitro transcription mRNA were synthesized from a PCR template containing a T7 promoter upstream followed by the codon-optimized open reading frame. All constructs contain a 5’ UTR, 3’ UTR and 125 polyA tail. All mRNA was transcribed using the HiScribe T7 High Yield RNA Synthesis Kit (New England Biolabs). Capping was cotranscriptionally performed using CleanCap Reagent AG (TriLink Biotechnologies). All uridine was replaced with N1-methylpseudouridine (TriLink Biotechnologies). In vitro transcription reaction was treated with DNase to eliminate the template, and dsRNA was removed by cellulose clean-up methods^38^. The final product was purified by sodium acetate precipitation.

### LNP formulation

LNPs were formulated as previously reported with modification^8^. A lipid mixture consisting of Dlin-MC3-DMA or SM102, DSPC (Avanti Polar Lipids), cholesterol (Sigma) and DMG-PEG_2000_ or DSPE-PEG_2000_ (Avanti Polar Lipids) was prepared in ethanol as a 20-mM stock. The molar composition used was 50:10:38.5:1.5 molar ratio. The lipid solution was mixed by flowing through a microfluidic mixing device Nanoassemblr (Precision Nanosystems) with an aqueous mRNA solution in 10-mM citrate buffer (pH 4) at a 1:3 organic to an aqueous volume ratio at a total flow rate of 4 ml per min. The resulting LNPs were then diluted twice with PBS (pH 7.4) immediately and further dialysed overnight. Then, the next day, LNPs were filtered through a 0.22-µm filter.

### LNP characterization

The size distribution, particle number per millilitre and mode size of LNP was measured using NanoSight NS300 (Malvern Panalytical). The zeta potential of LNPs was measured using a Zetasizer Nano ZS (Malvern Panalytical). Total mRNA content and encapsulation efficiency were determined by performing a standard RiboGreen (Thermo Fisher) assay.

### Molecular cloning of nanobody

Genes encoding the TP1107 sequence were synthesized as a gene fragment (Integrated DNA Technologies) for cloning into a pET His6 TEV LIC cloning vector^23^. Plasmids will be deposited to the Addgene repository.

### Single-domain antibody expression and purification

pET-TP1107 was co-transformed alongside pEVOl-pAzF into B-95.ΔA *E. coli*, which expresses the orthogonal machinery for the incorporation of azPhe in recognition of UAG codon during protein translation^25^. The B-95.ΔA *E. coli* strain is a unique expression vector in which 95 of its original UAG codons have been replaced along with the elimination of release factor 1 to facilitate the improved incorporation efficiency of azPhe^26^.

An overnight culture was inoculated into fresh Terrific Broth media with appropriate antibiotics and grown at 37 °C and shaking until the optical density, OD_600_, reached 0.7–1.0. The single-domain antibody (sdAb) expression was induced by the addition of IPTG (1 mM), l-arabinose (0.02%) and azPhe amino acid (2 mM). Protein expression was continued for a further 12–14 h at 30 °C before harvesting the bacteria by centrifugation. Bacterial pellets were harvested by centrifugation (4,000*g*, 20 min) and resuspended in Ni-NTA wash buffer followed by cell lysis using a high-pressure homogenizer (Avestin Emulsiflex C5).

On lysis, cell debris was centrifuged (12,000*g*, 30 min) and the supernatant was collected for purification via an immobilized metal affinity chromatography column. Additional size exclusion chromatography was used to remove non-specifically bound proteins using Superdex 75 10/300 GL gel filtration column (GE Healthcare). The sdAb concentration was determined using Nanodrop (Thermo) spectrophotometer at 280 nm.

### Negative-stain TEM

Negative-stain TEM was carried out by applying 3 µl of a 0.05 mg ml^−1^ solution onto a continuous carbon TEM grid (EMS 300 mesh), which was pretreated in a plasma chamber (30 s, 15-mA plasma current) followed by multiple applications of uranyl formate (0.01% w/v). Imaging was performed on a Thermo L120C TEM device at a magnification of 92k, yielding a physical pixel size of 1.55 Å per pixel. Here 41 images were recorded on a Ceta direct electron detector. Single-particle analysis was carried out in the RELION v. 3.1.2 software package^24^. Briefly, images had their CTF parameters estimated followed by automated particle picking and successive rounds of two-dimensional classification to homogenize the particle stack. This yielded 19k particles for ab initio three-dimensional (3D) model generation, which was carried out in cryoSPARC^39^ and further 3D refinements were finalized in RELION, resulting in an ~16-Å 3D reconstruction. A Protein Data Bank (PDB) model was generated by initially performing rigid-body fitting of the mouse IgG (PDB ID 1IGY (ref. ^40^)) using UCSF Chimera^41^. This fitted model then underwent a molecular dynamics flexible fitting refinement using UCSF ChimeraX/ISOLDE^42,43^ to fit the antibody into the 3D volume. The resultant PDB was then used as a template for HADDOCK docking^44–48^ of the nanobody. The docking clusters that best fit the experimental density were then considered for a further round of molecular dynamics flexible fitting with tight torsion and distance restraints based on the original 1IGY model and the starting model for the nanobody.

### Conjugation of TP1107 to DBCO-PEG_2000_-DSPE

Azide-incorporated TP1107_optimal_ can be directly conjugated onto DBCO-PEG_2000_-DSPE through Strain-Promoted Alkyne-Azide Cycloaddition (SPAAC) chemistry. The conjugation mixture was prepared at a DBCO:azide molar ratio of 2:1. Meanwhile, to illustrate the effect of randomly oriented sdAbs, 2 molar excess of NHS-azide (198 Da, Thermo) was initially conjugated onto TP1107 sdAb. Excess unconjugated NHS-azide linkers were removed using a 7 K MWCO Zeba desalting column (Thermo). The azide-modified/azide-incorporated sdAbs were mixed with DSPE-PEG_2000_-DBCO at a 0.5 molar excess and left for 24 h at 37 °C as TP1107_random_. No further purification was required.

### Post-insertion of active targeting module into LNPs and functionalized mAb-TP1107_optimal/random_ LNPs

The post-insertion of DSPE-PEG_2000_-TP1107 was performed by adding lipidated nanobody to the LNP solution at 0.5% w/w, followed by brief mixing before incubating at 4 °C for 48 h unmixed. The free TP1107 or unreacted DSPE-PEG_2000_-DBCO was removed via Amicon 100 kDa MWCO (Merck) ultrafiltration. A total of five washes (2,000 rpm, 10 min) were completed. Functionalized LNP was prepared by mixing antibodies with TP1107_optimal/random_ LNP at the selected ratio and incubated 4 °C overnight.

### Calculation of TP1107 per LNP

The sdAb number of each LNP calculated as below:

$${{\rm{Number}\; \rm{of}\; \rm{sdAbs}\; \rm{per}\; \rm{LNP}}}=\frac{{{\rm{Number}\; \rm{of}\; \rm{sdAbs}\; \rm{in}\; \rm{solution}}}}{{{\rm{Number}\; \rm{of}\; \rm{LNPs}\; \rm{in}\; \rm{solution}}}},$$

where the concentration of sdAb was calculated from western blot using JESS Simple Western (Bio-Techne) and the number of LNPs was measured by NanoSight NS300 (Malvern Panalytical).

### Targeting antibody

Targeting antibody used for in vitro and ex vivo studies is anti-hTfR (OKT9, purchased from WEHI), mouse anti-hCD3 antibody (UCHT1, Thermo Fisher), mouse anti-hCD4 (SK3, BioLegend), mouse anti-hCD5 (UCHT2, Thermo Fisher), mouse anti-hCD7 (124-1D1, Thermo Fisher), mouse anti-hCD22 (eBio4KB128 (4KB128), Thermo Fisher) and mouse IgG1 kappa isotype control (P3.6.2.8.1, Thermo Fisher). Targeting antibody used for the in vivo study is mouse anti-mouse CD3ε (QA17A05, BioLegend).

### Conjugation of mTfR to DSPE-PEG_2000_-DBCO

A 5 molar excess of NHS-azide was initially conjugated to purified mAb_TfR_ (OKT9), a kind gift from J. Mintern’s group. Excess unconjugated NHS-azide was removed by a 7K MWCO Zeba desalting column (Thermo Fisher) following the manufacturer’s protocol. The azide-mTfR was incubated with DSPE-PEG_2000_-DBCO at 37 °C overnight at a DBCO:azide ratio of 2:1. No further purification is required.

### Preparation of mAb_lysine_ LNPs

Roughly 0.05% w/w of mAb-PEG2000-DSPE mixture was added to the formulated LNPs. The reaction was incubated at 4 °C for 48 h. The post-inserted LNP was then concentrated by an ultrafiltration system and slowly applied to a 90-cm-bed-length gravity-flow size exclusion column prepared with Sepharose-CL4B gel^12,13^. The mobile phase was PBS. The fractions that contained LNP were collected and concentrated by the ultrafiltration system. The mRNA concentration was measured by a RiboGreen assay, as described before. The particle size was determined by NTA.

### Cell culture maintenance

Jurkat cells were maintained with RPMI media (Gibco) supplied with 10% foetal bovine serum (FBS) and penicillin/streptomycin (100 U ml^−1^). Cells were cultured at 37 °C in a humidified incubator with 5% atmospheric CO_2_ along with routine testing or mycoplasma contamination.

### Mouse models

B6.Cg-Gt(ROSA)26Sortm14(CAG-tdTomato)Hze/J (IMSR_JAX: 007914) mice were purchased from The Jackson Laboratory and maintained locally at the Monash animal research platform, and mice were ordered and shipped for the experiment on request. C57BL/6J mice were obtained from the Monash animal research platform. Male and female mice aged 6–14 weeks were used in the experiments. Ai14 mice were used for the data shown in Fig. 6 and Supplementary Fig. 14. C57BL/6J mice were used for the data shown in Supplementary Fig. 14. All the experimental procedures followed the protocols approved by the Institutional Animal Care and Use Committee at Monash University, and the experimental plan was approved by the Monash Office of Research Ethics and Integrity Committee under ethics approval nos. 37404 and 41587. All the mice groups were randomized and gender balanced.

### Human PBMC collection and purification

Healthy donors aged between 18 years and 50 years old in both sexes were recruited voluntarily after the invitation to participate. Ethics is approved by the Monash University Human Research Ethics Committee, application ID 37405. Human blood was collected as per the experimental plan. Here 10–30 ml of human blood was collected and diluted with PBS before carefully layered on Ficoll-Paque PLUS density gradient media at 1:1 v/v. A PBMC layer was collected after 400*g*, 40-min spin and washed with prewarmed RPMI media twice. PBMCs were either used for experiments or frozen in cell-freezing media at –80 °C.

### Antibody-capturing LNP safety assessment and cytokine measurement

C57BL/6 mice were acquired under ethics approval no. 37404. The mice received intravenous injections of 0.1 mg kg^−1^ of various LNP formulations or control groups, vehicle, unmodified SM102/DSPE LNP, unmodified SM102/DSPE LNP and mCD3 LNP with SM102 and DSPE-PEG_2000_. Blood samples were collected at 6 h and 24 h post-injection to evaluate the impact on liver enzymes (alanine transaminase and aspartate transaminase) and cytokine release. After 24 h, the animals were euthanized for liver and spleen harvesting and subsequent histological analysis. The harvested liver and spleen tissues were fixed in 10% neutral-buffered formalin for at least 48 h. Two mice from each group were assessed by a veterinary pathologist for further evaluation. Plasma was obtained by centrifuging blood samples at 1,000*g* for 5 min at 4 °C and stored as single aliquots. The levels of mouse cytokines IL-1α, IL-1β, IL-10, IL-6, MIP-1α, MCP-1, IL-2, TNF-α, IFN-γ and IL-4 were measured using the BD Cytometric Bead Array Mouse Flex Set according to the manufacturer’s protocol. Data collection was performed using a Stratedigm S1000EXi flow cytometer.

### Cytokine measurement for the whole blood stimulated with targeted LNP

Healthy donor’s blood was collected on the day of the experiment in heparin-coated collection tubes. Different targeted LNPs were added to each well at a final concentration of 2 ng μl^−1^ and incubated for 24 h. The blood samples were then centrifuged to collect plasma for cytokine measurement. Human cytokines IFN-γ, IL-1α, IL-1β and TNF were quantified using the BD Cytometric Bead Array Human Flex Set according to the manufacturer’s protocol. Data collection was performed using a Stratedigm S1000EXi flow cytometer.

### Cell association and transfection assay with functionalized LNPs

To assess the binding and transfection efficiencies of functionalized LNPs in Jurkat cells, approximately 50,000 or 100,000 cells were added to individual wells in a 96-well plate. A final concentration of 0.5 ng µl^−1^ or 1 ng µl^−1^ of mRNA was added to the cells and incubated at 37 °C for varied time periods. Subsequently, cells were washed three times with 2% FBS/PBS following centrifugation at 400*g* for 5 min. Cells were then resuspended in 80 µl of 2% FBS/PBS, and the MFI was quantified using a Stratedigm S1000EXi flow cytometer. eGFP and Cy5 fluorescence were excited at 488 nm and 642 nm, respectively, with fluorescence emission collected at 520/20 nm and 676/29 nm.

### Human PBMC association and transfection assay with functionalized LNPs

To assess the binding and transfection efficiencies of the functionalized LNPs, approximately 500,000 PBMCs were added to individual wells in a 96-well plate with functionalized LNPs at a final concentration of 1 ng µl^−1^. Then, cells were incubated at 37 °C for 24 h. Then, PBMCs were washed thrice with 2% FBS/PBS after centrifugation at 400*g* for 5 min. To phenotype the subpopulations, cells were stained against αCD3-PE mAb (clone OKT3, BioLegend), αCD4-BV510 mAb (clone OKT4, BioLegend), αCD8-BV786 mAb (clone SK1, BioLegend), αCD19-BV421 mAb (clone HIB19, BioLegend), αCD14-Alexa Fluor 700 mAb (clone HCD14, BioLegend), αCD56-BV605 mAb (clone 5.1H11, BioLegend) and viability dye (eBioscience Fixable Viability Dye eFluor 780, Thermo Fisher) on ice for 30 min. All the antibodies were used at 1:200 dilutions with Human TruStain FcX (BioLegend) as per the manufacturer’s protocol. After washing away the excessive antibody, cells were resuspended with 100 µl of 2% FBS/PBS for the flow analysis (Stratedigm S1000EXi). Cells were identified by a combination of surface markers: CD4^+^ T cells (CD3^+^ T cells and CD4^+^ T cells), CD8^+^ T cells (CD3^+^ T cells and CD8^+^ T cells), monocytes (CD3^−^, CD19^−^, CD56^−^ and CD14^+^), NK cells (CD3^−^, CD19^−^, CD14^−^ and CD56^+^) and B cells (CD3^−^ and CD19^+^). eGFP and Cy5 fluorescence was excited at 488 nm and 642 nm with fluorescence emission collected at 520/20 nm and 676/29 nm, respectively. Data in Supplementary Fig. 8 were obtained via a Cytek Aurora five-laser full spectrum cytometer.

### In vivo assessment of CD3 targeting of LNPs to T cells across multiple organs

Ai14 mice were injected intravenously with unmodified LNPs, CD3-targeted LNPs or isotype control LNPs loaded with Cre mRNA. After 24 h, blood was collected via cardiac puncture, and the mice underwent transcardiac perfusion with PBS to remove circulating blood. Red blood cells were lysed using ammonium–chloride–potassium buffer (Thermo Fisher) at a 1:10 (v/v) ratio twice, followed by washing with 2% FBS/PBS. The liver, spleen and lymph nodes (inguinal, iliac and cervical) were collected and processed as follows.

The liver was minced and digested using a gentleMACS dissociator with 2.8 mg ml^−1^ of collagenase H and 0.28 mg ml^−1^ of DNase. The digested mixture was filtered to remove the undigested material and subjected to a slow spin at 60*g*. The supernatant was collected and spun down to collect the pellet. The pellet was resuspended in 30% Percoll media and spun to remove hepatocytes, followed by resuspension with ammonium–chloride–potassium lysis buffer and washing with 2% FBS/Hanks’ balanced salt solution before antibody staining.

The spleen was minced with 1 mg ml^−1^ of collagenase III and 0.28 mg ml^−1^ of DNase and digested by constant, gentle mixing until fully digested. Cells were filtered and red blood cells were lysed using an ammonium–chloride–potassium buffer.

Lymph nodes were collected and homogenized by passing through a 0.45-µm filter. Dissociated cells were collected and washed with media.

All the immune cell pellets were stained with a flow cytometry panel containing the following antibodies: αCD3e-BV650 mAb (clone 145-2C11, BD Biosciences), αCD90.2-BV650 mAb (clone 53-2.1, BD Biosciences), αCD4-APC-Cy7 mAb (clone GK1.5, BioLegend), αCD8-BV711 mAb (clone 53-6.7, BD Biosciences), αCD19-BV786 mAb (clone 1D3, BD Biosciences), αCD11b-BV421 mAb (clone M1/70, BioLegend), αLy6C-BUV661 mAb (clone HK1.4.rMAb, BD Biosciences), αLy6G-BV605 mAb (clone 1A8, BD Biosciences), αCD45-Pacific Blue mAb (clone S18009F, BioLegend), αI-A/I-E-BV510 mAb (clone M5/114.15.2, BioLegend), αF4/80-PE/Dazzle mAb (clone BM8, BioLegend) and αCD11c-Alexa Fluor 700 mAb (clone N418, BioLegend). Additionally, Mouse BD Fc Block and viability dye (LIVE/DEAD Fixable Blue Dead Cell Stain Kit, Thermo Fisher) were included. Samples were incubated on ice for 30 min, followed by washing to remove excess antibody.

Flow cytometry was performed using a Cytek Aurora five-laser cytometer, and data were analysed using FlowJo v10.10.0 (BD Biosciences). Leucocyte phenotyping was conducted using the following markers: CD4^+^ T cells (CD45^+^, CD11b^−^, CD3e^+^ or CD4^+^); CD8^+^ T cells (CD45^+^, CD11b^−^, CD3e^+^ or CD8^+^); dendritic cells (CD45^+^, CD3e^−^, CD19^−^, CD11c^+^ or MHCII^+^); monocytes (CD45^+^, CD11b^+^, Ly6C^+^ or Ly6G^−^); neutrophils (CD45^+^ CD11b^+^, Ly6C^+^ or Ly6G^+^); macrophages (CD45^+^ CD11b^+^, Ly6C low, Ly6G^−^, F4/80^+^ or SSA low); and CD19^+^ B cells (CD45^+^, CD11b^−^, CD3^−^, CD19^+^ or MHCII^+^).

### Statistics and reproducibility

Data are presented as mean ± standard deviation (s.d.) based on the data obtained from at least *n* = 3 independent experiments, wells or mice. Statistical significance was determined using GraphPad Prism 9.0 and stated in each figure legend.

### Reporting summary

Further information on research design is available in the Nature Portfolio Reporting Summary linked to this article.

## Online content

Any methods, additional references, Nature Portfolio reporting summaries, source data, extended data, supplementary information, acknowledgements, peer review information; details of author contributions and competing interests; and statements of data and code availability are available at 10.1038/s41565-025-01954-9.

## Supplementary information

Supplementary Information Supplementary Tables 1–3, Figs. 1–16 and reference.

Reporting Summary

Supplementary Data 1 Source data for Supplementary Figures.

## Source data

Source Data Fig. 2 Statistical source data.

Source Data Fig. 3 Statistical source data.

Source Data Fig. 4 Statistical source data.

Source Data Fig. 5 Statistical source data.

Source Data Fig. 6 Statistical source data.

## Data Availability

Uncropped images are used in the figures. Source data are provided with this paper.
