# Supplementary material for: A versatile antibody capture system drives specific in vivo delivery of mRNA-loaded lipid nanoparticles
Source: Nat Nanotechnol. 2025 Aug 4;20(9):1273–84. doi: 10.1038/s41565-025-01954-9 (PMC12443633; doi:10.1038/s41565-025-01954-9)
Supplement: Supplementary file 1 — Supplementary Tables 1–3, Figs. 1–16 and reference. [file 41565_2025_1954_MOESM1_ESM.pdf]

nature nanotechnology

Supplementary information https://doi.org/10.1038/s41565-025-01954-9

A versatile antibody capture system drives specific in vivo delivery of mRNA-loaded lipid nanoparticles

In the format provided by the authors and unedited

Table of Contents

Supplementary Figures *2*

Supplementary Table 1. 2

Supplementary Table 2. 3

Supplementary Table 3. 4

Supplementary Figure 1. 5

Supplementary Figure 2. 6

Supplementary Figure 3. 7

Supplementary Figure 4. 8

Supplementary Figure 5. 9

Supplementary Figure 6. 10

Supplementary Figure 7. 11

Supplementary Figure 8. 12

Supplementary Figure 9. 13

Supplementary Figure 10. 14

Supplementary Figure 11. 15

Supplementary Figure 12. 16

Supplementary Figure 13. 17

Supplementary Figure 14. 18

Supplementary Figure 15. 19

Supplementary Figure 16. 20

Supplementary Figures

The summarized data of the formulated particles used in this study.

LNP	Ionizable lipid	PEG lipid	Nanobody-PEG_{2000}-DSPE addition	Size/zeta potential
MC3/DMG-LNP	DlinMC3DMA	DMG-PEG_{2000}	N/A	N/A
MC3/DSPE-LNP	DlinMC3DMA	DSPE-PEG_{2000}	N/A	83±1.5 nm/-7.5
SM102/DMG-LNP	SM102	DMG-PEG_{2000}	N/A	N/A
SM102/DSPE-LNP	SM102	DSPE-PEG_{2000}	N/A	49±2.2 nm
$TP1107_{optimal}$-MC3/DSPE-LNP	DlinMC3DMA	DSPE-PEG_{2000}	0.5%w/w $TP1107_{optimal}$-PEG_{2000}-DSPE	85±5.4 nm/-10.2
$TP1107_{random}$-MC3/DSPE-LNP	DlinMC3DMA	DSPE-PEG_{2000}	0.5%w/w $TP1107_{random}$-PEG2000-DSPE	92±8.3 nm/-9.9
$mTfR_{optimal}$-MC3-LNP	DlinMC3DMA	DSPE-PEG_{2000}	0.5%w/w $TP1107_{optimal}$ -PEG_{2000}-DSPE	85±4 nm
$mTfR_{random}$-MC3-LNP	DlinMC3DMA	DSPE-PEG_{2000}	0.5%w/w $TP1107_{random}$ -PEG_{2000}-DSPE	95±5.2 nm
$TP1107_{optimal}$-SM102-LNP	SM102	DSPE-PEG_{2000}	0.5%w/w $TP1107_{optimal}$ -PEG2000-DSPE	62±3.5 nm
$mTfR_{optimal}$-SM102-LNP	SM102	DSPE-PEG_{2000}	0.5%w/w $TP1107_{optimal}$ -PEG2000-DSPE	60±1.4 nm
mAb_{Lysine}-SM102-LNP	SM102	DSPE-PEG_{2000}	0.05%w/w $Antibody_{lysine}$ -PEG2000-DSPE *	58 ±2 nm

Supplementary Table 1. Detailed summary of the LNP formulation that were employed in this study including the composition of each formulation used in the study and relevant hydrodynamic diameter and zeta potential measurements. All LNPs were formulated with 50% ionizable lipid, 10% DSPC , 38.5% cholesterol and 1.5% PEG lipid. *The addition of antibody-lipid was followed to achieve the same level of antibody concentration per LNP as previous study[1]. All data are mean ± SD.

The summarized data of the encapsulation efficiency of formulated particles			
**LNP**	Encapsulation Efficiency %	SD	n
MC3/DSPE-LNP	94	2	3
TP1107$_{optimal}$-MC3/DSPE-LNP	97.33	1.5	3
TP1107$_{random}$-MC3/DSPE-LNP	95	4.4	3
SM102/DSPE-LNP	95.50	0.9	3
TP1107$_{optimal}$-SM102/DSPE-LNP	92.73	1.3	3

Supplementary Table 2. Summary of the encapsulation efficiency of different LNP formulations measured by Ribogreen assay. All data are mean ± SD. N represents independent repeats.

The quantification of TP1107optimal and TP1107random per LNP			
**TP1107 functionalized LNP**	Concentration of TP1107 mg/mL	Particle number per mL	TP1107 per LNP
$TP1107_{optimal}$ #1	6.09	8.82E+11	262
$TP1107_{optimal}$ #2	4.09	7.62E+11	179
$TP1107_{optimal}$ #3	5.58	9.51E+11	196
$TP1107_{optimal}$ #4	4.61	7.18E+11	215
$TP1107_{random}$ #1	5.28	3.06E+11	578
$TP1107_{random}$ #2	4.89	3.39E+11	483
$TP1107_{random}$ #3	2.6	8.70E+10	417
$TP1107_{random}$ #4	3.6	1.38E+11	577

Supplementary Table 3. Summary of the particle concentration and TP1107 concentration for 4 independent LNP formulations.

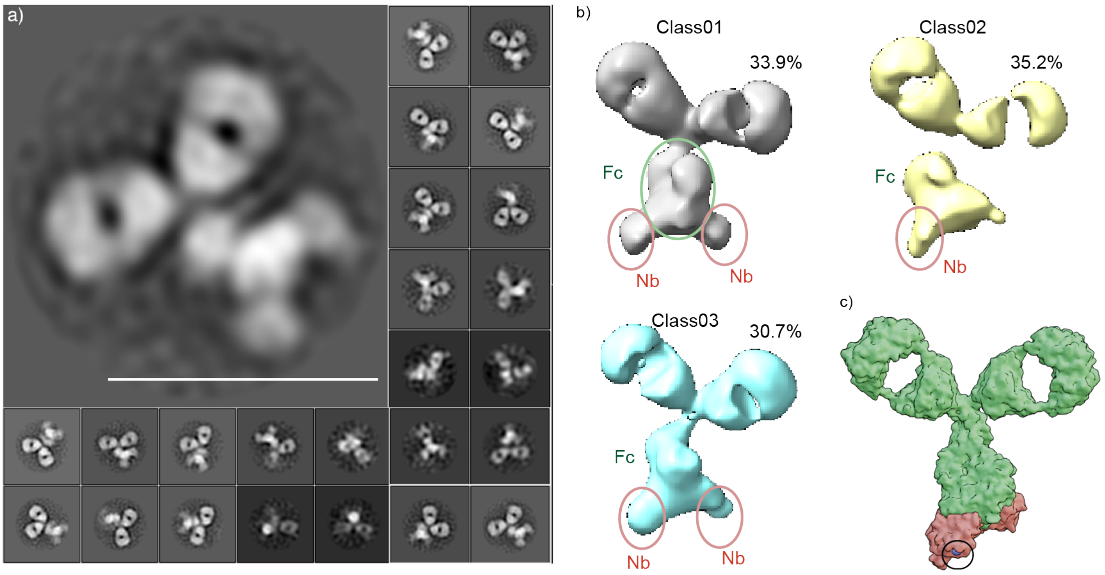

Supplementary Figure 1. Low resolution EM structure determination enables rational engineering of a nanobody to capture antibodies. a) TEM obtained for a, 2D projection of nanobody:antibody complexes. Scale bar is 200 Å. b) 3D models showing the nanobody binding to the Fc domain. c) 3D reconstruction of 2D projection from an initial rigid body fitting followed by docking of the nanobodies using HADDOCK. The Gln15 residue (highlighted in Blue and circled in black) was identified as a point of attachment to the LNP that would likely optimize the orientation of the subsequently captured antibody.

a

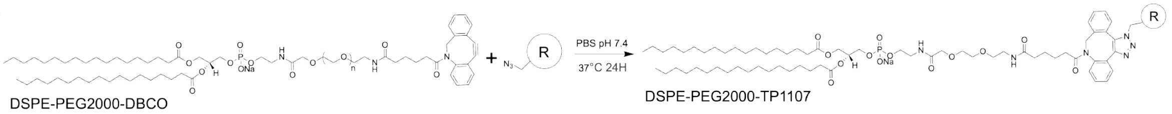

b

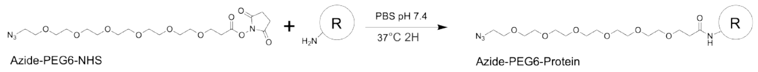

Supplementary Figure 2. The reaction scheme illustrates the click chemistry between TP1107-azide and DSPE-PEG2000-DBCO, as well as the conjugation of proteins (antibody or nanobody) with Azide-PEG6-NHS. a) The TP1107-azide and DSPE-PEG2000-DBCO reaction was conducted in PBS with a 2:1 DBCO molar ratio at 37°C for 24 hours, and the reaction mixture was subsequently stored at 4°C. b) Protein conjugation with Azide-PEG6-NHS was performed in PBS with a 5:1 Azide-PEG6-NHS molar ratio at 37°C for 2 hours. Unreacted Azide-PEG6-NHS was removed using a 7 kDa MWCO Zeba column."

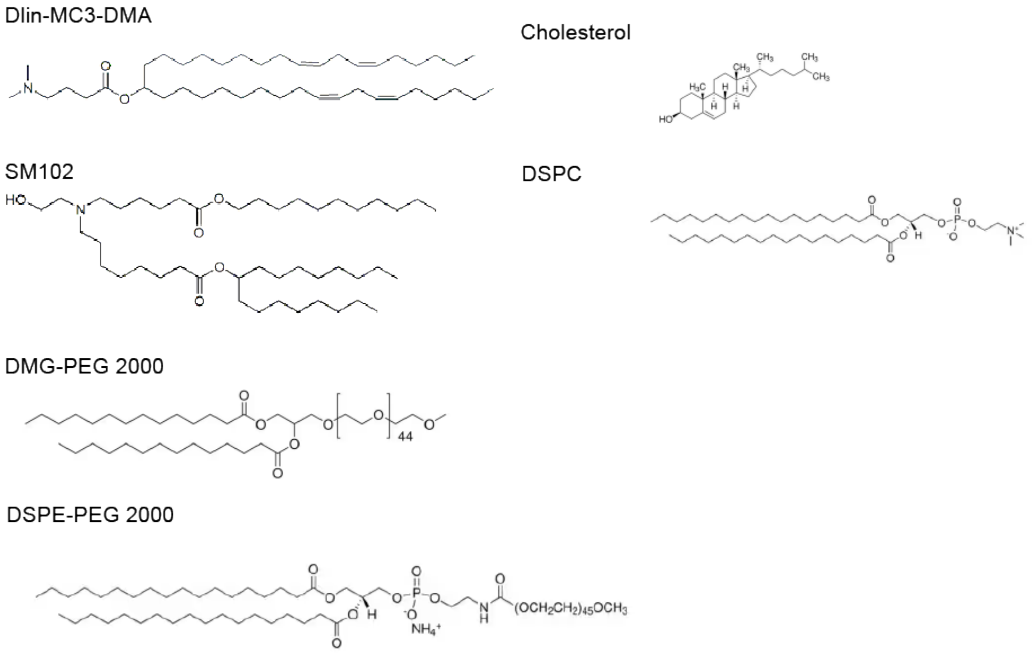

Supplementary Figure 3. Chemical structures of lipids utilized in this study, comprising two ionizable lipids (DLin-MC3-DMA [MC3] and SM102), a helper lipid (DSPC), a structural lipid (cholesterol), and two PEGylated lipids with distinct acyl chains (DMG-PEG2000 and DSPE-PEG2000).

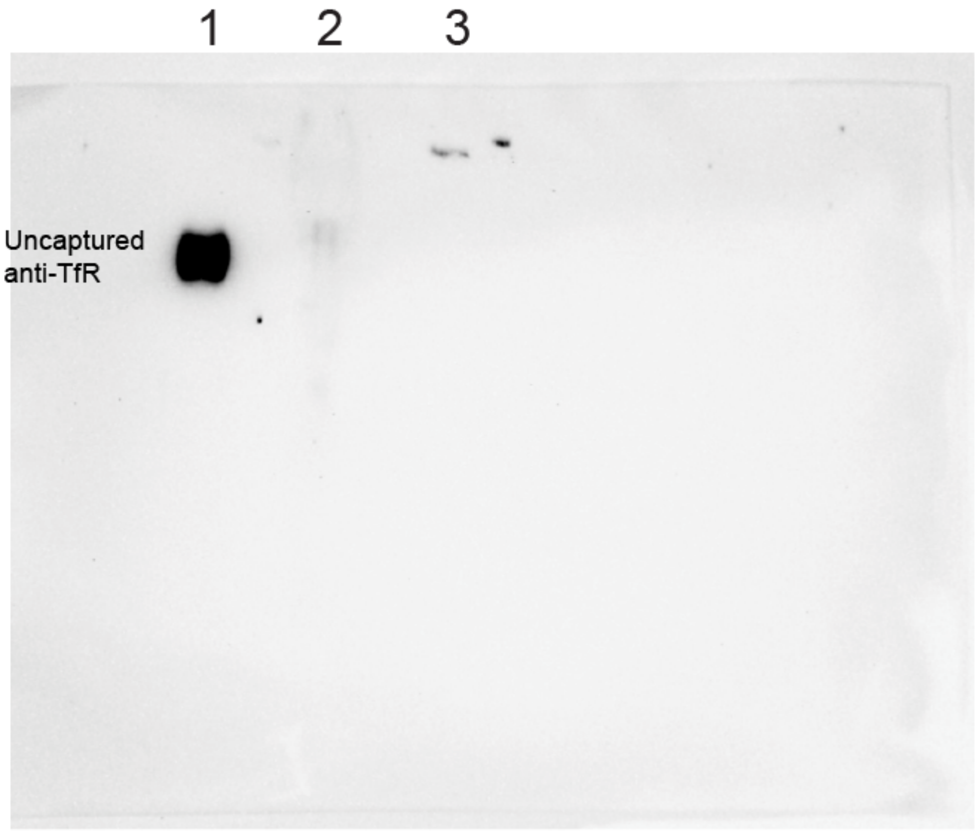

Supplementary Figure 4. LNP-TP1107$_{optimal}$ with anti-TfR antibody complex remained stable in human plasma. From left to right, sample loaded as following: Lane 1: 100ng of anti-TfR antibody, Lane 2: 100ng of anti-TfR captured on LNP-TP1107optimal and incubated with human plasma, Lane 3: 100ng of anti-TfR captured on LNP-TP1107optimal and incubated with PBS. The incubation period was 24 hours at 37oC. Protein gel was run under native conditions and followed by western blot detecting mouse antihuman TfR antibody. Only free anti-TfR can enter the gel, when anti-TfR is captured on the LNP it remains in the well. The absence of a band indicates there is no free anti-TfR in solution.

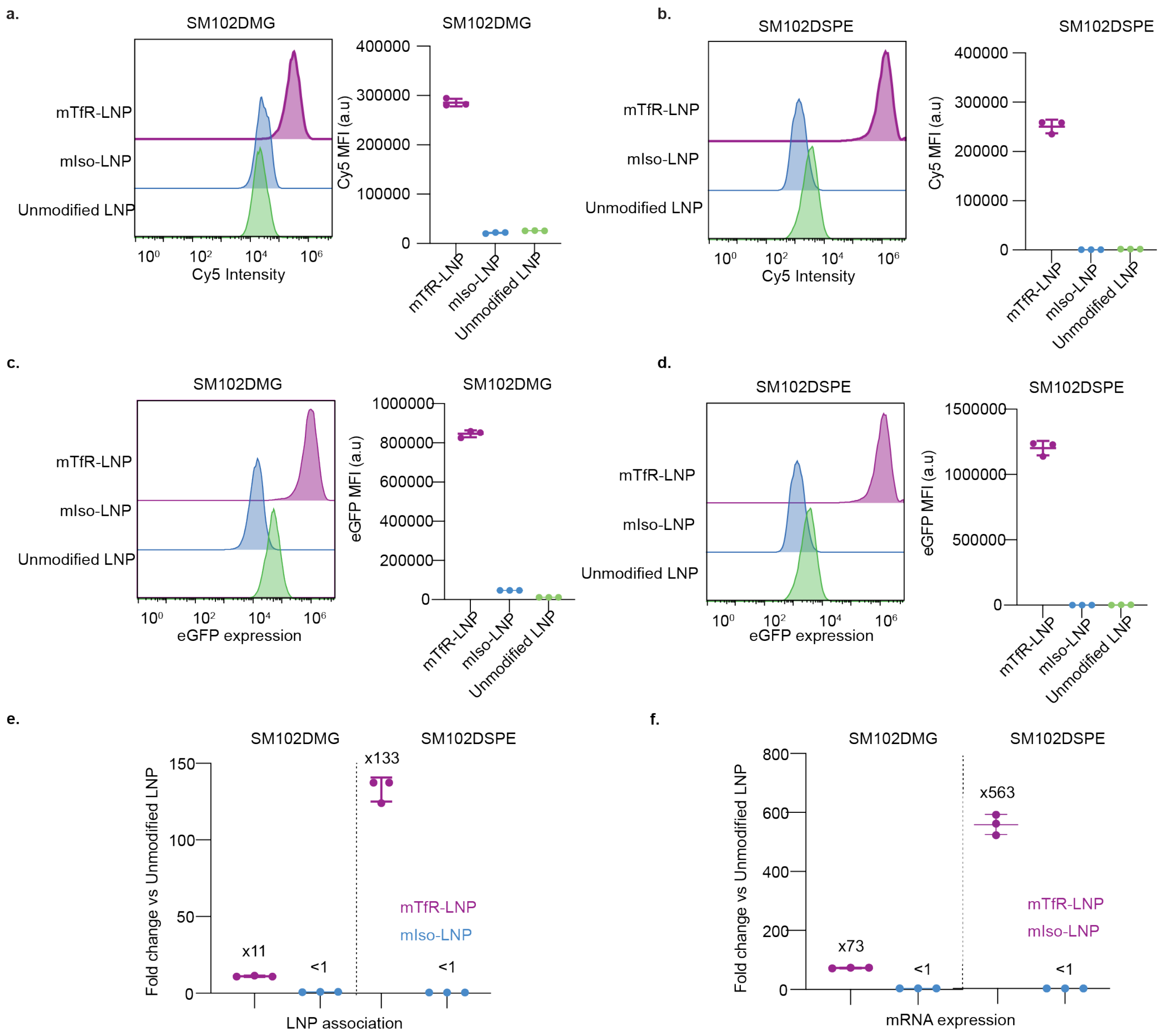

Supplementary Figure 5. TP1107-PEG2000-DSPE module with SM102-LNP showed enhanced cell binding and mRNA delivery with antiCD71 a) Cy5 mean fluorescence intensity (MFI) of Jurkat cells incubated with SM102/DMG-LNP and b) SM102/DSPE-LNP with either human TfR targeted LNP, isotype control and unmodified LNP. c) eGFP expression level of Jurkat cells incubated with SM102/DMG-LNP and d) SM102/DSPE-LNP with either human TfR targeted LNP, isotype control and unmodified LNP. e) calculated fold change of Cy5 MFI and f) eGFP MFI between mIso-LNP and either SM102/DMG-LNP or SM102/DSPE-LNP. Data represents mean ± SD (n = 3 replicate wells).

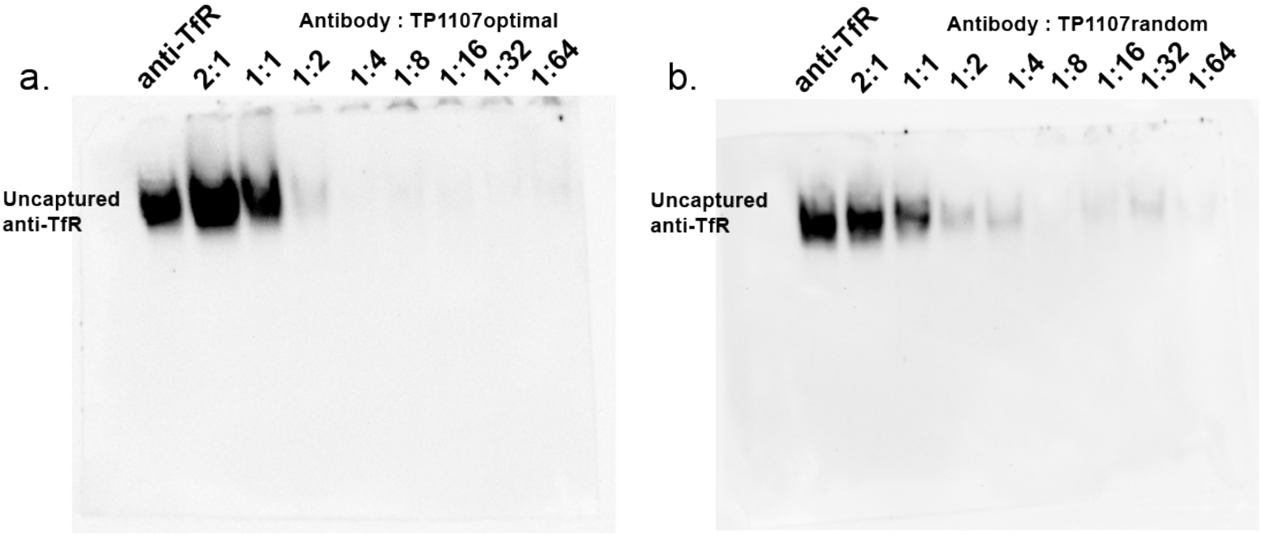

Supplementary Figure 6. TP1107$_{optimal}$-LNP successfully captured all antibodies in solution with 1:2 antibody vs TP1107$_{optimal}$ or lower ratio. From left to right, sample loaded as following: 100ng anti-TfR antibody, 2:1, 1:1, 1:2, 1:4, 1:8, 1:16, 1:32, and 1:64 of antibody (100ng) vs TP1107$_{optimal}$ or TP1107$_{random}$. Protein gel was run under native conditions and followed by western blot detecting mouse antihuman TfR antibody. Uncrop blots were shown in the figure.

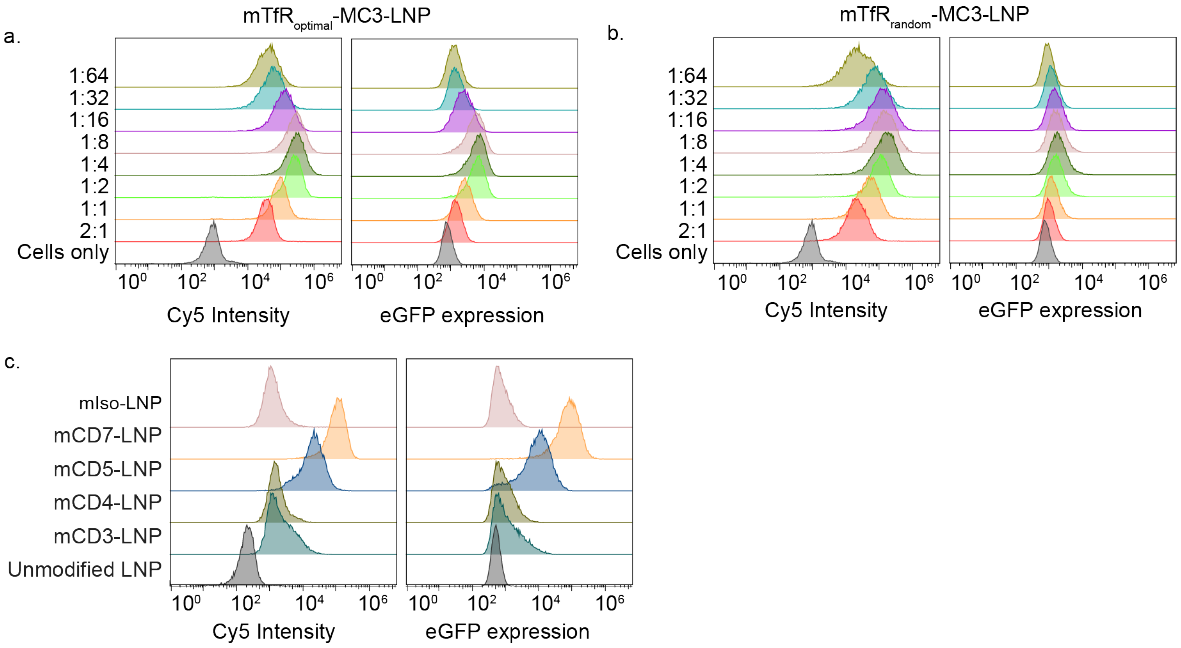

Supplementary Figure 7. Count histogram of conditions presented in Fig. 4. Cells were incubated with (a) TP1107 $_{optimal}$-MC3-LNP or (b) TP1107 $_{random}$-MC3-LNP at varying antibody vs TP1107 ratios (from 64:1 to 1:2) for 4 hours with 0.5 ng/µL mRNA. (c) Cells were treated with different LNP formulations for 24 hours."

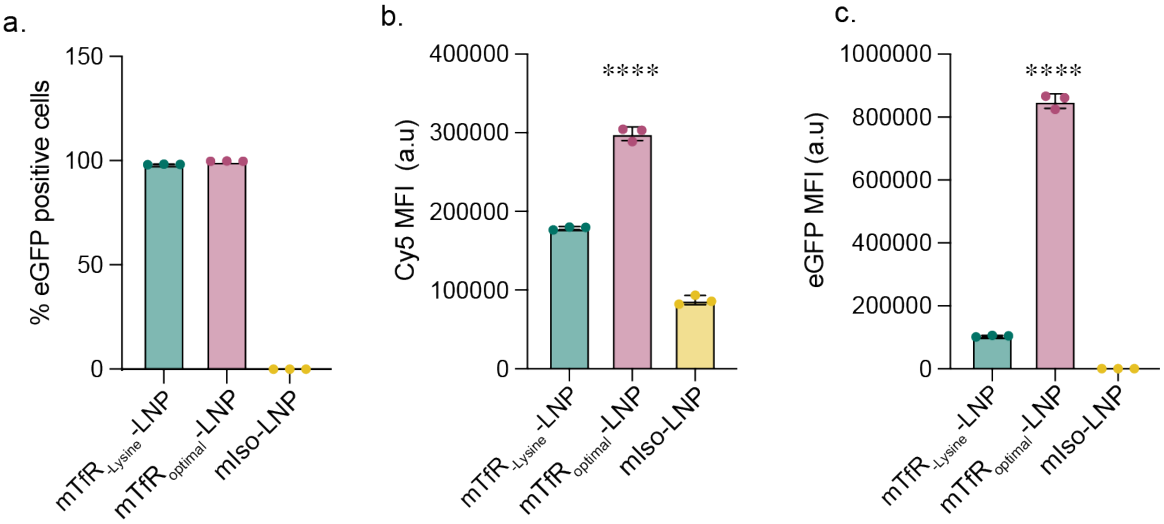

Supplementary Figure 8. TP1107 captured antibodies outperformed the conventional labelled antibodies on LNP over 24 hours. a) percentage of eGFP positive cells, b) Cy5 MFI and c) eGFP MFI, of Jurkat cells incubated with $mTfR_{Lysine}$-LNP reacted or $mTfR_{optimal}$-LNP for 24 hours at 0.5 ng/μL mRNA concentration. LNPs were formulated with SM102 as ionizable lipids. P value calculated by one-way ANOVA with post hoc Tukey's test. ****P<0.0001. All data represented as mean ± SD ((n = 3 replicate wells).).

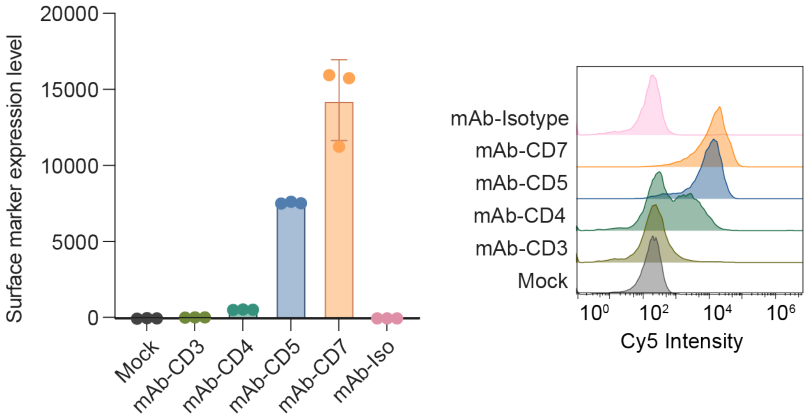

Supplementary Figure 9. The expression level of CD2, CD3, CD4, CD5 and CD7 on Jurkat cells. Jurkat cells were incubated with antibodies at 1:100 dilution for 2 hours at 4°C. Cells were washed with cold PBS three times before staining with goat anti-mouse Alexa Fluor 647 at 1:1000 dilution for 1 hour at 4°C. The mean fluorescent intensity (MFI) of Cy5 was collected and analyzed as surface marker expression level. Mock sample representative cells were only incubated with the secondary antibody. Count histogram of each sample was plotted on the right. All data are represented as mean ± SD ((n = 3 replicate wells).).

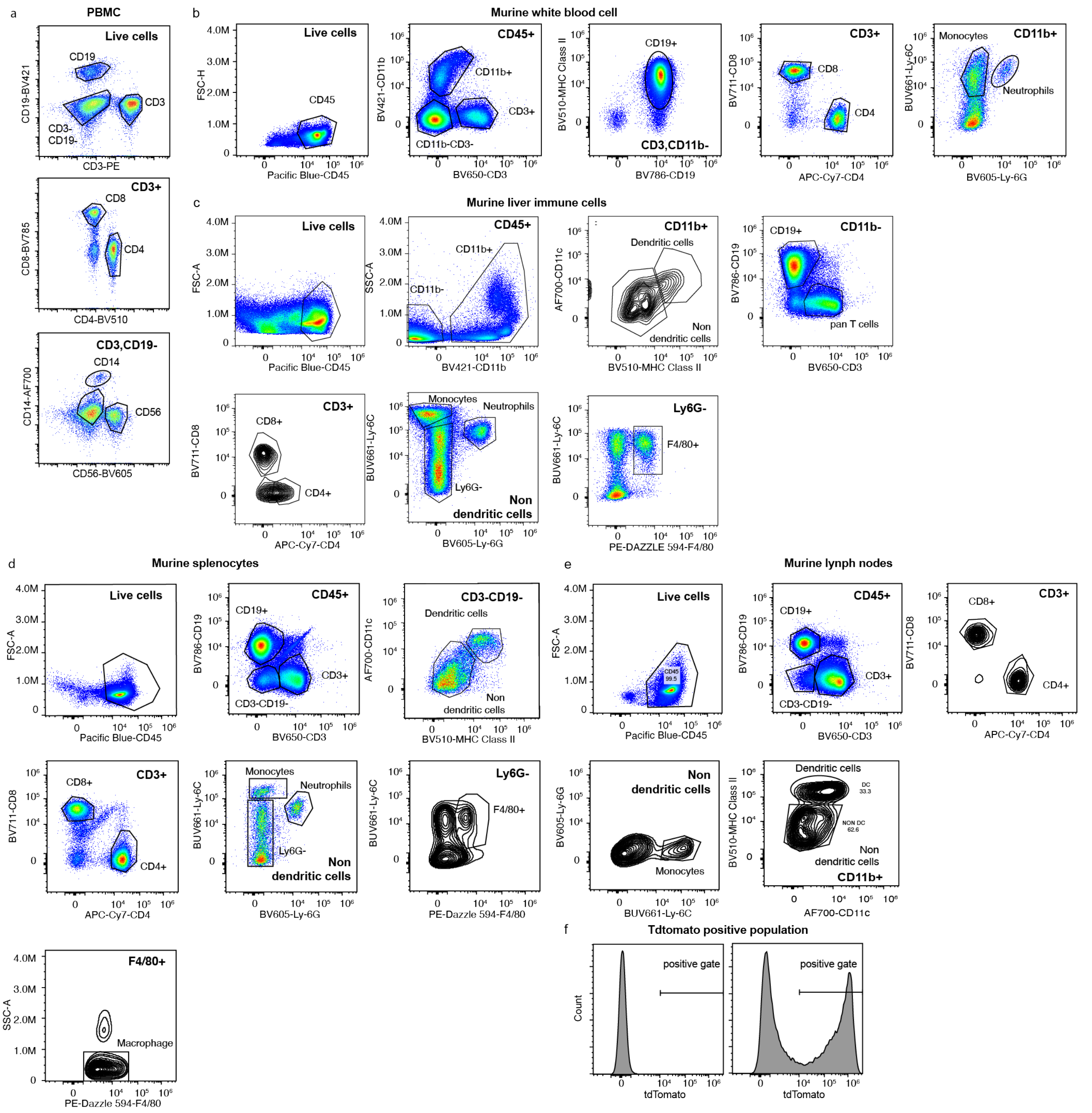

Supplementary Figure 10. Gating strategy of identifying subpopulation in purified human PBMC, murine white blood cells, murine splenocytes, murine liver immune cell population, and murine lymph node immune cell population. a) Purified human PBMC were stained with an antibody cocktail (Live and Dead dye, antiCD3, antiCD19, antiCD4, antiCD8a, antiCD14 and antiCD56). Live cells were selected first then subphenotyped into different groups as shown in the figure. **b)** enriched mice WBC, c), enriched murine liver immune cells d) enriched murine splenocytes immune cells and e) enriched murine lymph nodes immune cells were stained with an antibody cocktail (Live and Dead dye, antiCD45, antiCD3/antiCD90.2, antiCD19, antiCD4, antiCD8a, antiMHCII, antiCD11c, antiF4/80, antiLy6C and antiLy6G). f) an example of the gating strategy of tdTomato positive cells that was reported in the Figure 6. The cell population was selected based on FSC-A vs SSC-A and followed by selecting single cells based on FSC-A vs FSC-H. Live cells were selected as the negative population of the live/dead dye.

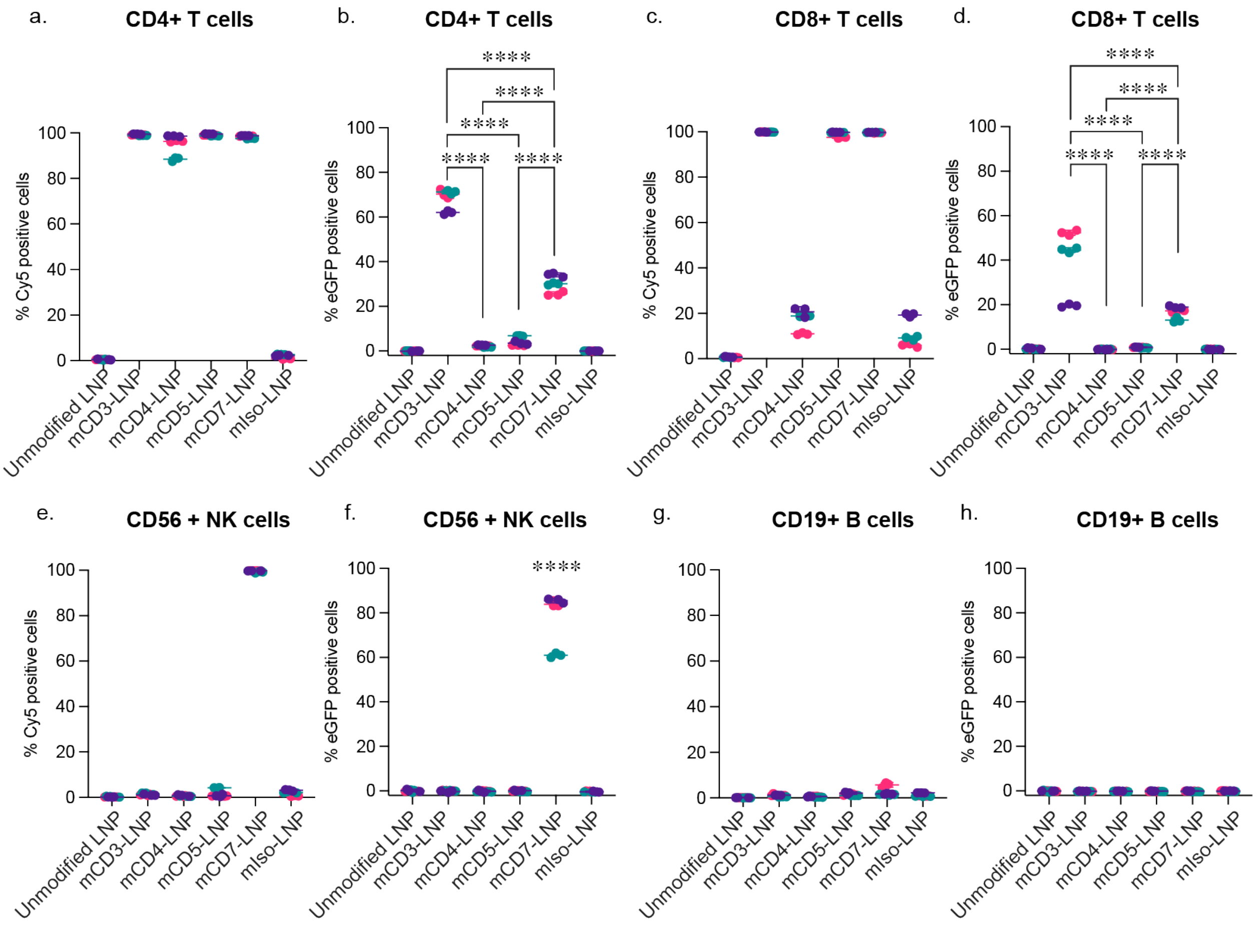

Supplementary Figure 11. The plotting of percentage of LNP association cells (Cy5 positive population,) and the percentage of EGFP expressing cells (eGFP positive population) with different immune cell population that were treated with mAb-LNP. The dataset is an extension of Figure 6. a and b, represented CD4+ T cell, c and d, represented CD8+ T cells population, e and f, represented CD56+ NK cell population g and h, represented CD19+ B cell population. Cluster dot represents individual donors (depicted by colours green, pink and purple). ****P < 0.0001; two-way ANOVA and Tukey's post-test (Compare row means - main row effects). All data are means ± SD; n = 3 independent donors.

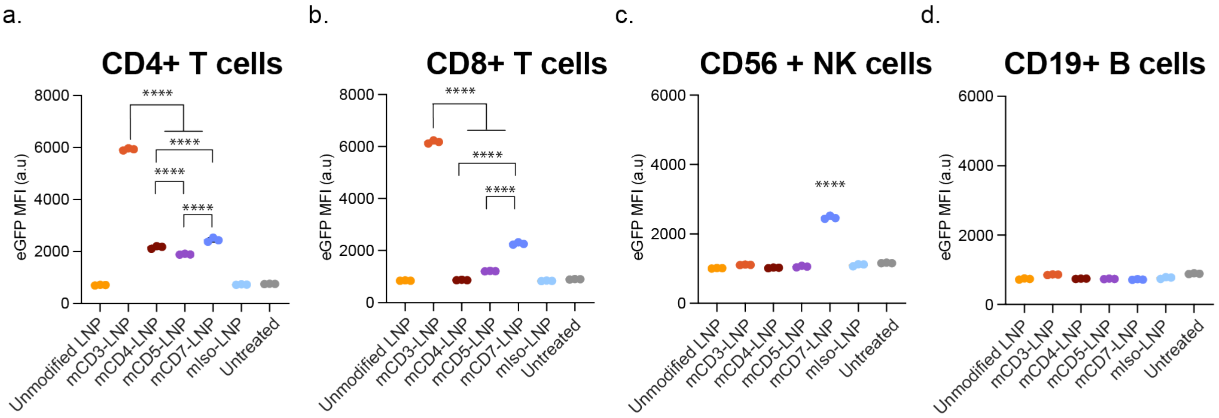

Supplementary Figure 12. The eGFP expression MFI of different immune population in human PBMC treated with different targeted LNP with SM102 as the ionizable lipid. a, CD4+ T cell, b, CD8+ T cells population, c, CD56+ NK cell population d, CD19+ B cell population. ****P < 0.0001; one-way ANOVA and Tukey's post-test. All data are means ± SD; (n = 3 replicate wells).

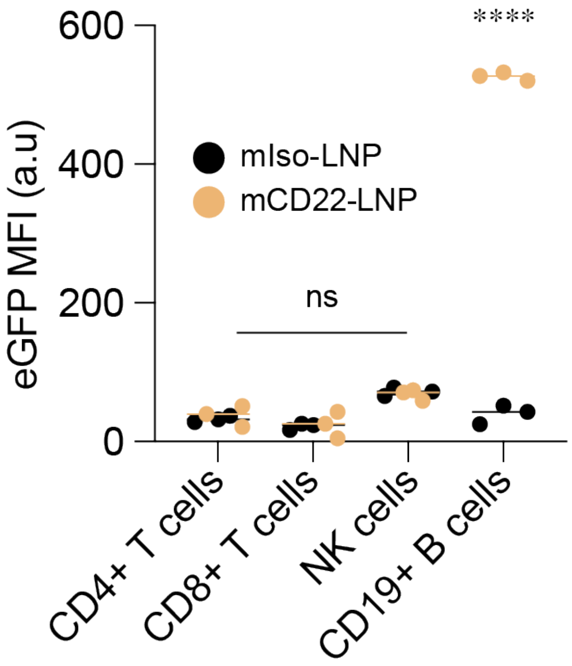

Supplementary Figure 13. The eGFP expression MFI of different immune population in human PBMC treated with CD22 targeted LNP. human PBMC cells were purified from fresh donated blood and incubated with either mCD22 LNP or the isotype control at 2ng/uL for 24 hours. eGFP expression was plotted. ****P < 0.0001; one-way ANOVA and Tukey's post-test. All data are means ± SD; n = 3 (n = 3 replicate wells).

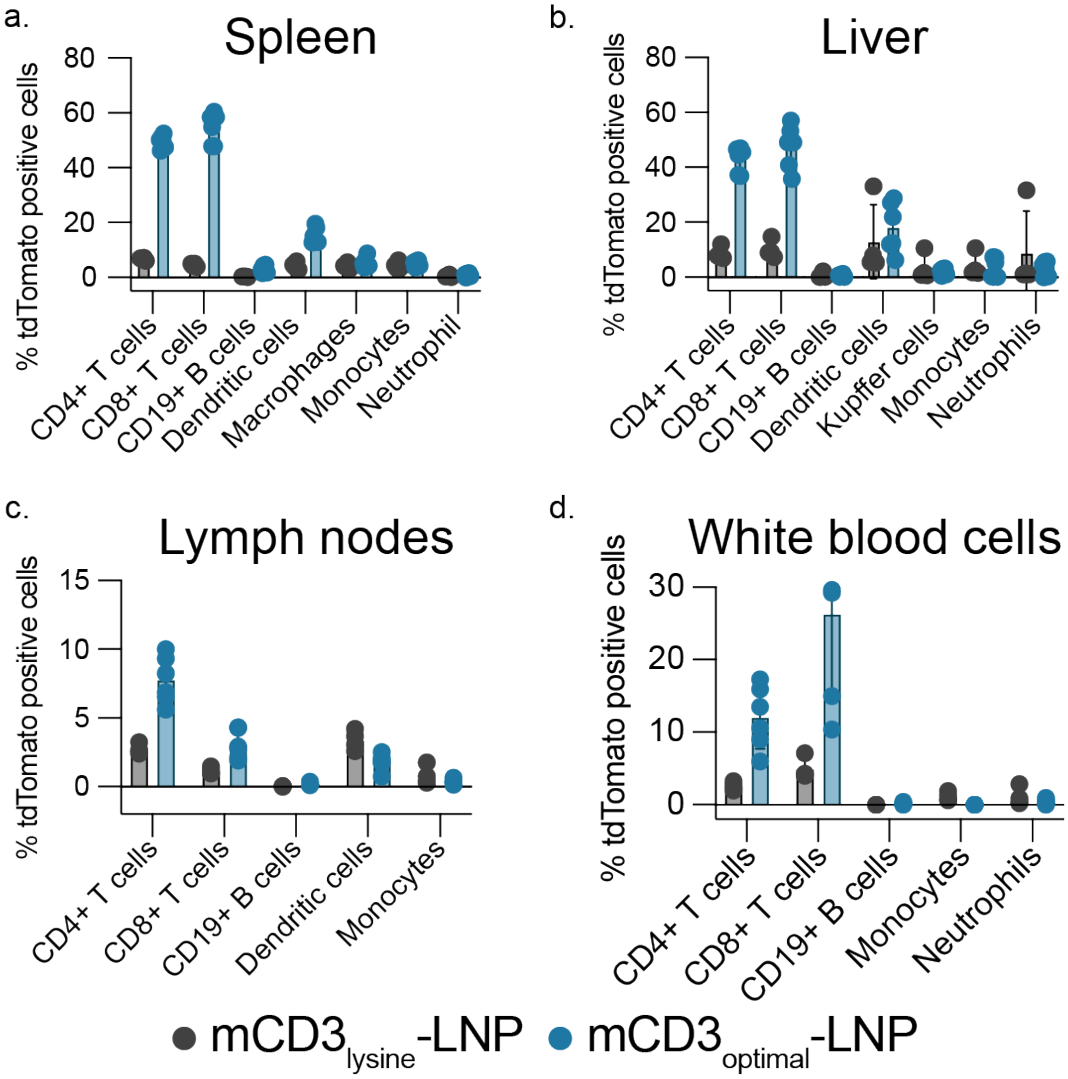

Supplementary Figure 14. mCD3$_{lysine}$ LNP transfected less tdTomato positive T cells in various organs compared to mCD3$_{optimal}$ LNP. mCD3$_{optimal}$ LNP (data from Figure 6) and mCD3$_{lysine}$ LNP was given 0.1mg/kg with Cre mRNA intravenously. Immune cells were harvested from a) spleen, b) liver, c) lymph nodes and d)circulating blood after 24 hours. The stained cells were analyzed by flow cytometry to identify the percentage of tdTomato positive cells in each sub population (CD4 + T cell, CD8 + positive T cell, dendritic cells, CD19 + B cells, monocytes, macrophage/Kupffer cells, and neutrophils.) n = 6 mice in two separate cohorts for mCD3$_{optimal}$ LNP (data from Figure 6) and n = 4 mice for mCD3$_{lysine}$ LNP group. All data are means and error bars represent SD.

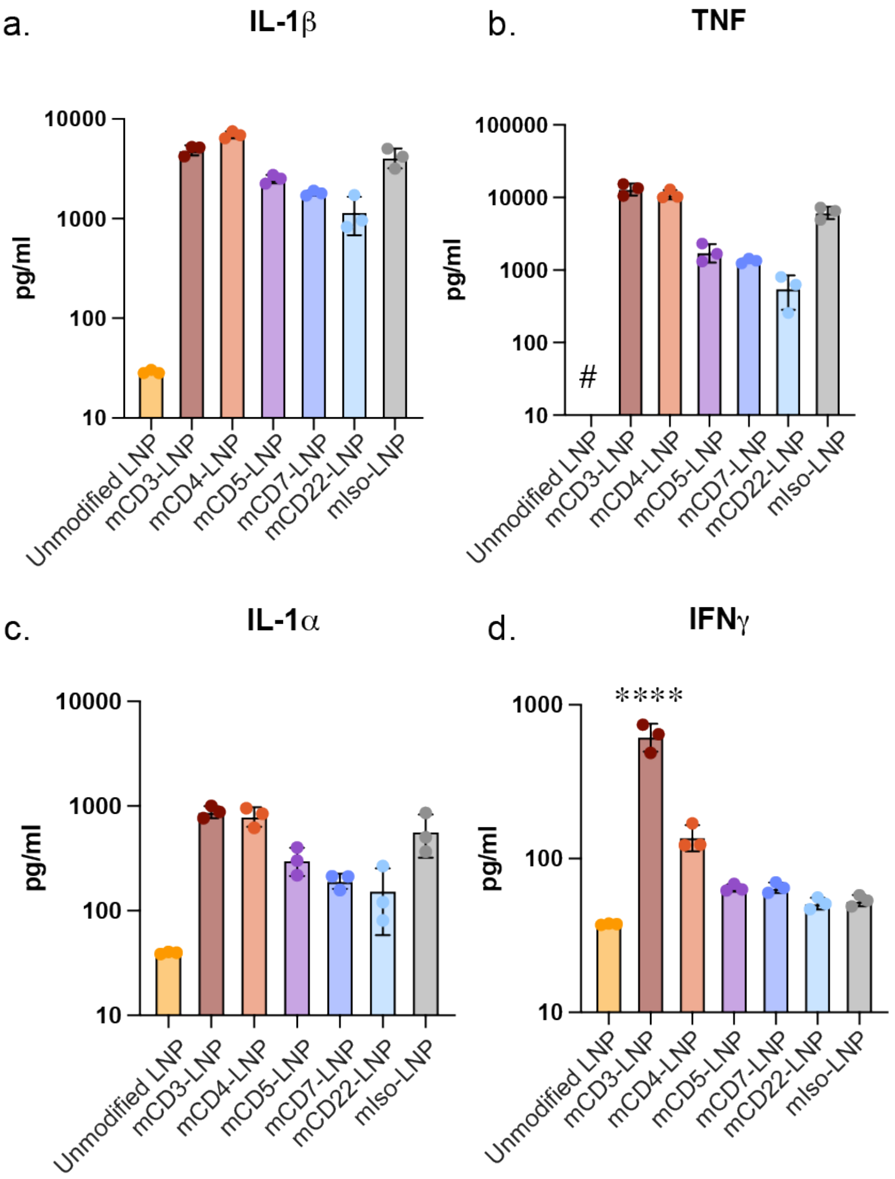

Supplementary Figure 15. CD3 targeted LNP promoted cytokine release contributed by T lymphocytes activation. Human whole blood was treated with 2ng/mL of different targeted LNP for 24 hours. Plasma was collected and a) IL-1b, b) TNF, c) IL-1a and d) IFNg were measured using Cytometric Bead Array (CBA) from BD biosciences following manufacture's instruction. Detection limit (10pg/ml). # below detection limit, ****P < 0.0001; one-way ANOVA and Dunnett's post-test. All data are means ± SD; (n = 3 replicate wells)..

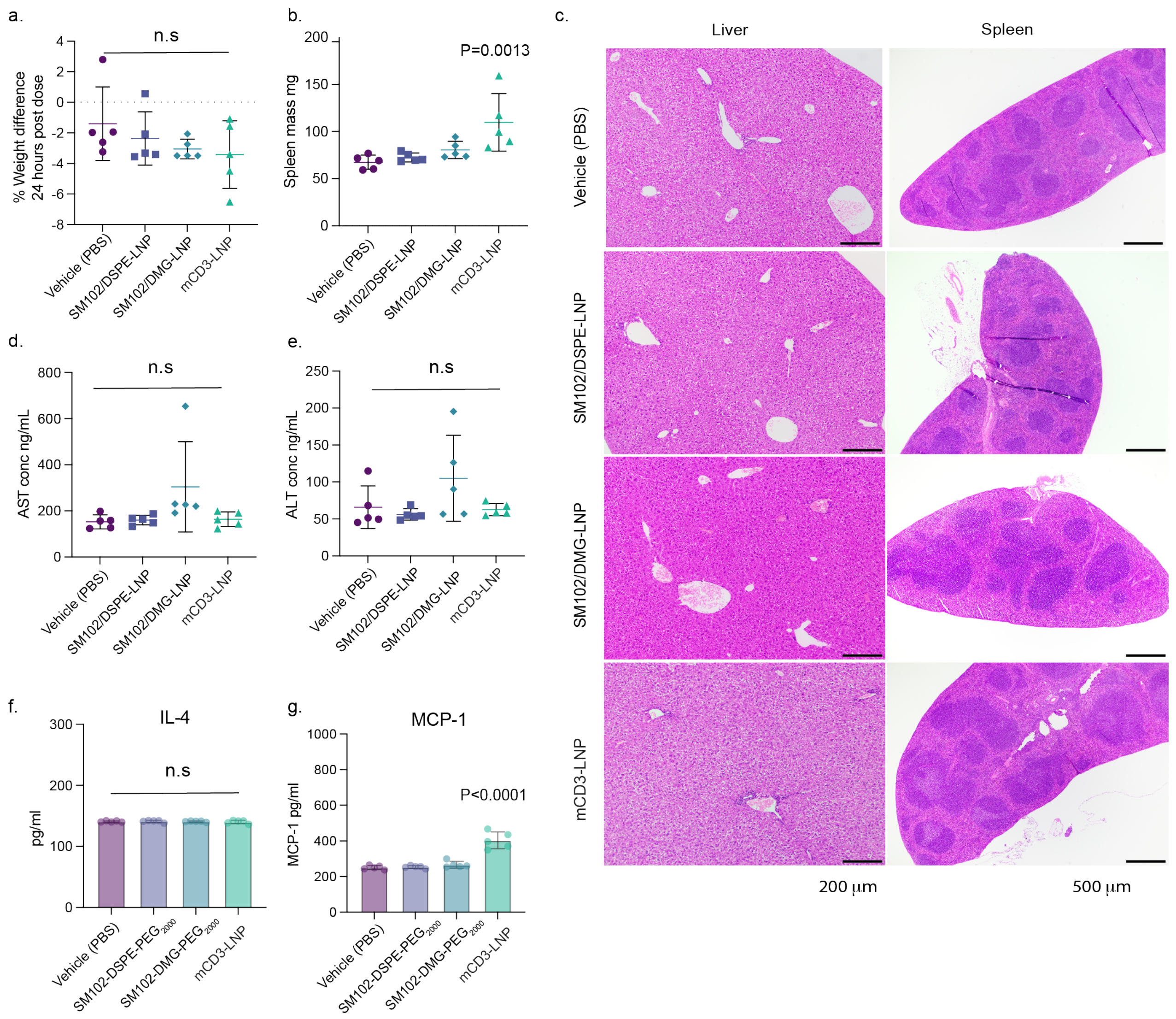

Supplementary Figure 16. mAb-CD3-LNP did not induce obvious safety issue compared with vehicle, SM102/DSPE-LNP and the current delivery formulation SM102/DMG-LNP. a) Mice weight changes, b) spleen weight, c) histopathology examination of liver and spleen samples, d) and e) ALT/AST plasma concentration at 6 hours post dosing, f) and g) cytokines profile at 24 hours post dosing were measured and performed to ensure the safety aspect of the targeting system. Two mice of each group were sent for pathology examination with randomized order. P value was calculated by one-way ANOVA and Dunnett's multiple comparison post-test (compared to the vehicle group). All data are means ± SD; n = 5 individual mouse for figure a, b, d, e and f.

Supplementary Reference

1. Tombácz, I., et al., *Highly efficient CD4+ T cell targeting and genetic recombination using engineered CD4+ cell-homing mRNA-LNPs.* Molecular Therapy, 2021. **29**(11): p. 3293-3304.
