# Supplementary material for: A versatile antibody capture system drives specific in vivo delivery of mRNA-loaded lipid nanoparticles
Source: Nat Nanotechnol. 2025 Aug 4;20(9):1273–84. doi: 10.1038/s41565-025-01954-9 (PMC12443633; doi:10.1038/s41565-025-01954-9)
Supplement: Supplementary file 2 — Reporting Summary [file 41565_2025_1954_MOESM2_ESM.pdf]

nature portfolio

Corresponding author(s): Angus Johnston

Last updated by author(s): Feb 24, 2025

Reporting Summary

Nature Portfolio wishes to improve the reproducibility of the work that we publish. This form provides structure for consistency and transparency in reporting. For further information on Nature Portfolio policies, see our Editorial Policies and the Editorial Policy Checklist.

Statistics

For all statistical analyses, confirm that the following items are present in the figure legend, table legend, main text, or Methods section.

n/a	Confirmed	
☐	☒	The exact sample size (*n*) for each experimental group/condition, given as a discrete number and unit of measurement
☐	☒	A statement on whether measurements were taken from distinct samples or whether the same sample was measured repeatedly
☐	☒	The statistical test(s) used AND whether they are one- or two-sided<br>*Only common tests should be described solely by name; describe more complex techniques in the Methods section.*
☐	☒	A description of all covariates tested
☐	☐	A description of any assumptions or corrections, such as tests of normality and adjustment for multiple comparisons
☐	☒	A full description of the statistical parameters including central tendency (e.g. means) or other basic estimates (e.g. regression coefficient) AND variation (e.g. standard deviation) or associated estimates of uncertainty (e.g. confidence intervals)
☐	☒	For null hypothesis testing, the test statistic (e.g. *F*, *t*, *r*) with confidence intervals, effect sizes, degrees of freedom and *P* value noted<br>*Give P values as exact values whenever suitable.*
☒	☐	For Bayesian analysis, information on the choice of priors and Markov chain Monte Carlo settings
☒	☐	For hierarchical and complex designs, identification of the appropriate level for tests and full reporting of outcomes
☒	☐	Estimates of effect sizes (e.g. Cohen's *d*, Pearson's *r*), indicating how they were calculated

Our web collection on statistics for biologists contains articles on many of the points above.

Software and code

Policy information about availability of computer code

Data collection	No code and software used.
Data analysis	We used GraphPad Prism, Flowjo V10 for flow cytometry analysis. For capillary immunoassay to determine protein concentration, we used Compass for SW. For data in Sup Fig 1, we used UCSF ChimeraX/iSOLDE .

For manuscripts utilizing custom algorithms or software that are central to the research but not yet described in published literature, software must be made available to editors and reviewers. We strongly encourage code deposition in a community repository (e.g. GitHub). See the Nature Portfolio guidelines for submitting code & software for further information.

Data

Policy information about availability of data

All manuscripts must include a data availability statement. This statement should provide the following information, where applicable:

- Accession codes, unique identifiers, or web links for publicly available datasets
- A description of any restrictions on data availability
- For clinical datasets or third party data, please ensure that the statement adheres to our policy

All data are available in the main manuscript or the supplementary materials. Requests for materials should be addressed to M.Z.C and A.J.

Research involving human participants, their data, or biological material

Policy information about studies with human participants or human data. See also policy information about sex, gender (identity/presentation), and sexual orientation and race, ethnicity and racism.

Reporting on sex and gender	All participants are aged between 18-50 years old with both sexes.
Reporting on race, ethnicity, or other socially relevant groupings	The ethnicity and other information is not collected.
Population characteristics	The information is not collected.
Recruitment	The recruitment was voluntary.
Ethics oversight	The experimental plan was approved by Monash University Human Research Ethics Committee, application ID 37405.

Note that full information on the approval of the study protocol must also be provided in the manuscript.

Field-specific reporting

Please select the one below that is the best fit for your research. If you are not sure, read the appropriate sections before making your selection.

☒ Life sciences ☐ Behavioural & social sciences ☐ Ecological, evolutionary & environmental sciences

For a reference copy of the document with all sections, see nature.com/documents/nr-reporting-summary-flat.pdf

Life sciences study design

All studies must disclose on these points even when the disclosure is negative.

Sample size	The sample size calculation is performed in this study. For in vitro and ex vivo assay, the n is reported in each figure legend accordingly based based on the magnitude and consistency of measurable differences between groups.<br><br>Previous studies and preliminary data obtained within our group which determine nanoparticle association with immune cells have formed the basis of group size.<br>A power analysis has been simulated to detect a moderate effect size and yields a required group size of 6.<br>Power=0.8<br>Alpha error probability= 0.05<br>Effect size= 1.6 (to determine a difference between means of 0.4 where the standard error is 0.25)<br>Sample size calculated= 6 mice per group.
Data exclusions	There is no data exclusion.
Replication	The replication is successful.
Randomization	For the animal study, mice have been randomized while receiving treatment and balanced between males and females.
Blinding	For this study, blinding is not applicable because the administration of treatment and data collection was done by the same person.

Reporting for specific materials, systems and methods

We require information from authors about some types of materials, experimental systems and methods used in many studies. Here, indicate whether each material, system or method listed is relevant to your study. If you are not sure if a list item applies to your research, read the appropriate section before selecting a response.

Materials & experimental systems

n/a	Involved in the study
☐	☒ Antibodies
☐	☒ Eukaryotic cell lines
☐	☐ Palaeontology and archaeology
☐	☒ Animals and other organisms
☐	☐ Clinical data
☐	☐ Dual use research of concern
☐	☐ Plants

Methods

n/a	Involved in the study
☒	☐ ChIP-seq
☐	☒ Flow cytometry
☒	☐ MRI-based neuroimaging

Antibodies

Antibodies used

Targeting antibody: Targeting antibody that used in in vitro and ex vivo study is anti-hTfR (OKT9, purchased from WEHI facility, Victoria, Australia), mouse anti-hCD3 Antibody (UCHT1, Thermo Fisher), mouse anti-hCD4 (SK3, Biolegend), Mouse anti-hCD5 (UCHT2, Thermo Fisher), mouse anti-hCD7 (124-1D1, Thermo Fisher) and Mouse IgG1 kappa Isotype Control (P3.6.2.8.1, Thermo Fisher). Targeting antibody that used in in vivo study is mouse anti-mouse CD3ε (QA17A05, Biolegend) and Mouse IgG1 kappa Isotype Control (P3.6.2.8.1, Thermo Fisher).

Human panel: αCD3-PE mAb (clone OKT3, Biolegend), αCD4-BV510 mAb (clone OKT4, Biolegend), αCD8-BV785 mAb (clone SK1, Biolegend), αCD19-BV421 mAb (clone HIB19, Biolegend), αCD14-Alexa Fluor 700 mAb (clone HCD14, Biolegend), αCD56-BV605 mAb (clone 5.1H11, Biolegend),

Mouse panel: αCD3e-BV650 mAb (clone 145-2C11, BD Biosciences), αCD90.2-BV650 mAb (clone 53-2.1, BD Biosciences), αCD4-APC-Cy7 mAb (clone GK1.5, BioLegend), αCD8-BV711 mAb (clone 53-6.7, BD Biosciences), αCD19-BV786 mAb (clone 1D3, BD Biosciences), αCD11b-BV421 mAb (clone M1/70, BioLegend), αLy-6C-BUV661 mAb (clone HK1.4.rMAb, BD Biosciences), αLy-6G-BV605 mAb (clone 1A8, BD Biosciences), αCD45-Pacific Blue mAb (clone S18009F, BioLegend), αI-A/I-E-BV510 mAb (clone M5/114.15.2, BioLegend), αF4/80-PE/Dazzle mAb (clone BM8, BioLegend), and αCD11c-Alexa Fluor 700 mAb (clone N418, BioLegend). Additionally, Mouse BD Fc Block™ and viability dye (LIVE/DEAD™ Fixable Blue Dead Cell Stain Kit, Thermo Fisher) were included.

Validation

The antibodies that used in this study are commercially validated and commercially tested.

Eukaryotic cell lines

Policy information about cell lines and Sex and Gender in Research

Cell line source(s)

Jurkat cell line was obtained from ATCC and maintained in our lab.

Authentication

We have purchased the Jurkat cell line from ATCC.

Mycoplasma contamination

Cell lines are routinely tested for mycoplasma contamination and are negative.

Commonly misidentified lines
(See ICLAC register)

NO commonly misidentified line was used in this study.

Palaeontology and Archaeology

Specimen provenance

Provide provenance information for specimens and describe permits that were obtained for the work (including the name of the issuing authority, the date of issue, and any identifying information). Permits should encompass collection and, where applicable, export.

Specimen deposition

Indicate where the specimens have been deposited to permit free access by other researchers.

Dating methods

If new dates are provided, describe how they were obtained (e.g. collection, storage, sample pretreatment and measurement), where they were obtained (i.e. lab name), the calibration program and the protocol for quality assurance OR state that no new dates are provided.

☐ Tick this box to confirm that the raw and calibrated dates are available in the paper or in Supplementary Information.

Ethics oversight

Identify the organization(s) that approved or provided guidance on the study protocol, OR state that no ethical approval or guidance was required and explain why not.

Note that full information on the approval of the study protocol must also be provided in the manuscript.

Animals and other research organisms

Policy information about studies involving animals; ARRIVE guidelines recommended for reporting animal research, and Sex and Gender in Research

Laboratory animals	B6.Cg-Gt(ROSA)26Sortm14(CAG-tdTomato)Hze/J mice, IMSR_JAX:007914. C57BL/6Jmice was obtained from Monash Animal research platform . Animals are housed under the following condition: 12h light/12h dark cycle, 22-25C and 35-37% humidity.
Wild animals	This study did not involve wild animals.
Reporting on sex	In the animal study, both sexes were used and balanced.
Field-collected samples	The study did not involve samples collected from the field.
Ethics oversight	All the experimental procedures followed the protocols approved by the Institutional Animal Care and Use Committee at Monash University and experimental plan was approved by the Monash Office of Research Ethics and Integrity committee under ethics 37404 or 41587.

Note that full information on the approval of the study protocol must also be provided in the manuscript.

Clinical data

Policy information about clinical studies

All manuscripts should comply with the ICMJE guidelines for publication of clinical research and a completed CONSORT checklist must be included with all submissions.

Clinical trial registration	*Provide the trial registration number from ClinicalTrials.gov or an equivalent agency.*
Study protocol	*Note where the full trial protocol can be accessed OR if not available, explain why.*
Data collection	*Describe the settings and locales of data collection, noting the time periods of recruitment and data collection.*
Outcomes	*Describe how you pre-defined primary and secondary outcome measures and how you assessed these measures.*

Dual use research of concern

Policy information about dual use research of concern

Hazards

Could the accidental, deliberate or reckless misuse of agents or technologies generated in the work, or the application of information presented in the manuscript, pose a threat to:

No	Yes	
☒	☐	Public health
☒	☐	National security
☒	☐	Crops and/or livestock
☒	☐	Ecosystems
☒	☐	Any other significant area

Experiments of concern

Does the work involve any of these experiments of concern:

No	Yes	
☒	☐	Demonstrate how to render a vaccine ineffective
☒	☐	Confer resistance to therapeutically useful antibiotics or antiviral agents
☒	☐	Enhance the virulence of a pathogen or render a nonpathogen virulent
☒	☐	Increase transmissibility of a pathogen
☒	☐	Alter the host range of a pathogen
☒	☐	Enable evasion of diagnostic/detection modalities
☒	☐	Enable the weaponization of a biological agent or toxin
☒	☐	Any other potentially harmful combination of experiments and agents

Plants

Seed stocks

Report on the source of all seed stocks or other plant material used. If applicable, state the seed stock centre and catalogue number. If plant specimens were collected from the field, describe the collection location, date and sampling procedures.

Novel plant genotypes

Describe the methods by which all novel plant genotypes were produced. This includes those generated by transgenic approaches, gene editing, chemical/radiation-based mutagenesis and hybridization. For transgenic lines, describe the transformation method, the number of independent lines analyzed and the generation upon which experiments were performed. For gene-edited lines, describe the editor used, the endogenous sequence targeted for editing, the targeting guide RNA sequence (if applicable) and how the editor was applied.

Authentication

Describe any authentication procedures for each seed stock used or novel genotype generated. Describe any experiments used to assess the effect of a mutation and, where applicable, how potential secondary effects (e.g. second site T-DNA insertions, mosiacism, off-target gene editing) were examined.

Flow Cytometry

Plots

Confirm that:

☒ The axis labels state the marker and fluorochrome used (e.g. CD4-FITC).

☒ The axis scales are clearly visible. Include numbers along axes only for bottom left plot of group (a 'group' is an analysis of identical markers).

☒ All plots are contour plots with outliers or pseudocolor plots.

☒ A numerical value for number of cells or percentage (with statistics) is provided.

Methodology

Sample preparation

The human blood was collected upon experimental plan. human blood was collected and diluted with PBS before carefully layered on Ficoll-Paque PLUS density gradient media with 1:1 v/v. PBMC layer was collected after 400g, 40 mins spin and washed with prewarmed RPMI media twice. PBMCs were either used for experiments or frozen in cell frozen media in -80oC for future use.

To assess the binding and transfection efficiency of the functionalized LNPs approximately 500,000 PBMC were added to individual wells in a 96-well plate with functionalized LNPs at 1ng/µL final concentration. Then cells were incubated at 37oC for 24 hours. PBMC then were washed thrice with 2% FBS-PBS after centrifugation at 400x g for 5 minutes. To phenotype the sub-populations, cells were stained against αCD3-PE mAb (clone OKT3, Biolegend), αCD4-BV510 mAb (clone OKT4, Biolegend), αCD8-BV786 mAb (clone SK1, Biolegend), αCD19-BV421 mAb (clone HIB19, Biolegend), αCD14-Alexa Fluor 700 mAb (clone HCD14, Biolegend), αCD56-BV605 mAb (clone 5.1H11, Biolegend), and viability dye (eBioscience™ Fixable Viability Dye eFluor™ 780, Thermofisher) on ice for 30 min. Antibodies were all used at 1:200 dilutions with Human TruStain FcX™ (Biolegend) as manufacturer's protocol. After washing away the excessive antibody, cells were resuspended with 100 ? L 2% FBS-PBS for the flow analysis (Stratedigm S1000EXi). Cells were identified by a combination of surface markers: CD4+ T cells (CD3+ and CD4+), CD8+ T cells (CD3+ and CD8+), monocytes (CD3-, CD19-, CD56-, and CD14+), NK cells (CD3-, CD19-, CD14- and CD56+), and B cells (CD3– and CD19+). eGFP and Cy5 fluorescence was excited at 488 and 642 nm with fluorescence emission collected at 520/20 nm and 676/29 nm respectively.

For in vivo assay. Ai14 mice were injected intravenously with unmodified lipid nanoparticles (LNPs), CD3-targeted LNPs, or isotype control LNPs loaded with Cre mRNA. After 24 hours, blood was collected via cardiac puncture, and the mice underwent transcardiac perfusion with PBS to remove circulating blood. Red blood cells were lysed using ACK buffer (Thermo Fisher, USA) at a 1:10 (v/v) ratio twice, followed by washing with 2% FBS-PBS. The liver, spleen, and lymph nodes (inguinal, iliac, and cervical) were collected and processed as follows:

The liver was minced and digested using a gentleMACS™ dissociator with 2.8 mg/mL Collagenase H and 0.28 mg/mL DNase. The digested mixture was filtered to remove undigested material and subjected to a slow spin at 60g. The supernatant was collected and spun down to collect the pellet. The pellet was resuspended in 30% Percoll media and spun to remove hepatocytes, followed by resuspension with ACK lysis buffer and washing with 2% FBS HBSS before antibody staining.

The spleen was minced with 1 mg/mL Collagenase III and 0.28 mg/mL DNase and digested by constant gentle mixing until fully digested. Cells were filtered and red blood cells lysed using ACK buffer.

Lymph nodes were collected and homogenized by passing through a 0.45 µm filter. Dissociated cells were collected and washed with media.

All immune cell pellets were stained with a flow cytometry panel containing the following antibodies: αCD3e-BV650 mAb (clone 145-2C11, BD Biosciences), αCD90.2-BV650 mAb (clone 53-2.1, BD Biosciences), αCD4-APC-Cy7 mAb (clone GK1.5, BioLegend), αCD8-BV711 mAb (clone 53-6.7, BD Biosciences), αCD19-BV786 mAb (clone 1D3, BD Biosciences), αCD11b-BV421 mAb (clone M1/70, BioLegend), αLy-6C-BUV661 mAb (clone HK1.4.rMAb, BD Biosciences), αLy-6G-BV605 mAb (clone 1A8, BD Biosciences), αCD45-Pacific Blue mAb (clone S18009F, BioLegend), αI-A/I-E-BV510 mAb (clone M5/114.15.2, BioLegend), αF4/80-PE/Dazzle mAb (clone BM8, BioLegend), and αCD11c-Alexa Fluor 700 mAb (clone N418, BioLegend). Additionally, Mouse BD Fc Block™ and viability dye (LIVE/DEAD™ Fixable Blue Dead Cell Stain Kit, Thermo Fisher) were included. Samples were incubated on ice for 30 minutes, followed by washing to remove excess antibody.

Flow cytometry was performed using a Cytek Aurora 5 laser cytometer, and data were analyzed using FlowJo (BD Biosciences). Leukocyte phenotyping was conducted using the following markers: CD4+ T cells (CD45+, CD11b-, CD3e+, CD4+); CD8+ T cells (CD45+, CD11b-, CD3e+, CD8+); dendritic cells (CD45+, CD3e-, CD19-, CD11c+, MHCII+); monocytes (CD45+, CD11b+, Ly6C+, Ly6G-); neutrophils (CD45+ CD11b+, Ly6C+, Ly6G+); macrophages (CD45+ CD11b+, Ly6C low, Ly6G-, F4/80+, SSA low); and CD19+ B cells (CD45+, CD11b-, CD3-, CD19+ or MHCII+).

Instrument	Stratedigm S1000EXi flow cytometer, Cytek Aurora 5 laser cytometer.
Software	Flowjo V10 for analysis.
Cell population abundance	No sorting has been done in this study.
Gating strategy	For in vitro experiments, all samples were gated using forward scatter and side scatter to identify events corresponding to cells, and then using forward scatter height vs. area to enrich for single cells, next alive cells were selected by negativity for viability dye.<br>For sex vivo and in vivo amples, all samples were initially gated using forward scatter and side scatter to identify events corresponding to cells, and then using forward scatter height vs. area to enrich for single cells, next alive cells were selected by negativity for viability dye. The follow gating steps are presented in principal and supplementary figures.

☒ Tick this box to confirm that a figure exemplifying the gating strategy is provided in the Supplementary Information.
